# Pathogenic variants in the human m^6^A reader *YTHDC2* are associated with primary ovarian insufficiency

**DOI:** 10.1172/jci.insight.154671

**Published:** 2022-03-08

**Authors:** Sinéad M. McGlacken-Byrne, Ignacio Del Valle, Polona Le Quesne Stabej, Laura Bellutti, Luz Garcia-Alonso, Louise A. Ocaka, Miho Ishida, Jenifer P. Suntharalingham, Andrey Gagunashvili, Olumide K. Ogunbiyi, Talisa Mistry, Federica Buonocore, GOSgene, Berta Crespo, Nadjeda Moreno, Paola Niola, Tony Brooks, Caroline E. Brain, Mehul T. Dattani, Daniel Kelberman, Roser Vento-Tormo, Carlos F. Lagos, Gabriel Livera, Gerard S. Conway, John C. Achermann

**Affiliations:** 1 Genetics and Genomic Medicine, UCL Great Ormond Street Institute of Child Health, University College London, London, United Kingdom.; 2 Department of Paediatric Endocrinology, Great Ormond Street Hospital for Children National Health Service (NHS) Foundation Trust, London, United Kingdom.; 3 EGA Institute for Women’s Health, University College London, London, United Kingdom.; 4 GOSgene, Genetics and Genomic Medicine, UCL Great Ormond Street Institute of Child Health, University College London, London, United Kingdom.; 5 Department of Molecular Medicine and Pathology, University of Auckland, Auckland, New Zealand.; 6 Laboratory of Development of the Gonads, Genetic Stability Stem Cells and Radiations, CEA/IBFJ/iRCM, University of Paris-Saclay, University Paris City, Fontenay aux Roses, France.; 7 Wellcome Sanger Institute, Wellcome Genome Campus, Hinxton, Cambridge, United Kingdom.; 8 Department of Histopathology, Great Ormond Street Hospital for Children NHS Foundation Trust, London, United Kingdom.; 9 Developmental Biology and Cancer Department, UCL Great Ormond Street Institute of Child Health, University College London, London, United Kingdom.; 10 UCL Genomics, Zayed Centre for Research, University College London, London, United Kingdom.; 11 Chemical Biology & Drug Discovery Lab, School of Pharmaceutical Chemistry, Faculty of Medicine and Science, San Sebastián University, Providencia, Santiago, Chile.

**Keywords:** Endocrinology, Genetics, Genetic diseases, Molecular genetics, Reproductive biochemistry

## Abstract

Primary ovarian insufficiency (POI) affects 1% of women and carries significant medical and psychosocial sequelae. Approximately 10% of POI has a defined genetic cause, with most implicated genes relating to biological processes involved in early fetal ovary development and function. Recently, *Ythdc2*, an RNA helicase and N6-methyladenosine reader, has emerged as a regulator of meiosis in mice. Here, we describe homozygous pathogenic variants in *YTHDC2* in 3 women with early-onset POI from 2 families: c. 2567C>G, p.P856R in the helicase-associated (HA2) domain and c.1129G>T, p.E377*. We demonstrated that *YTHDC2* is expressed in the developing human fetal ovary and is upregulated in meiotic germ cells, together with related meiosis-associated factors. The p.P856R variant resulted in a less flexible protein that likely disrupted downstream conformational kinetics of the HA2 domain, whereas the p.E377* variant truncated the helicase core. Taken together, our results reveal that *YTHDC2* is a key regulator of meiosis in humans and pathogenic variants within this gene are associated with POI.

## Introduction

Primary ovarian insufficiency (POI) affects 1% of women and is characterized by early loss of normal ovarian function and depletion of the ovarian reserve (1). POI is diagnosed in women who present before the age of 40 with estrogen deficiency, amenorrhea for at least 4 months, and evidence of raised follicle-stimulating hormone of more than 40 IU/L on 2 occasions (2). While most women with POI present with secondary amenorrhea, approximately 10% present with primary amenorrhea with or without absent puberty (3). POI is a condition that is associated with significant medical and psychosocial consequences (3). In a minority of women, the etiology is easily identified, with POI occurring secondary to ovarian damage arising from chemotherapy, radiotherapy, autoimmunity, or environmental agents (4). X chromosome abnormalities, such as Turner and fragile X syndromes, account for up to 5% of cases (5). Many of the remaining affected women may have an underlying genetic cause. This hypothesis is supported by the high incidence of familial POI, the increased incidence of POI within consanguineous pedigrees, and the association of POI with multisystem syndromes (6–9). Obtaining a specific genetic diagnosis allows targeted genetic screening and counseling, which is particularly relevant for gene mutations that carry an associated malignancy risk (e.g., *BRCA2*, *MCM9*) (10, 11). In recent years, next-generation sequencing has implicated variants in approximately 60 genes in the molecular pathogenesis of POI (6, 8). Further genes have been implicated in female fertility using transgenic mouse models. This, along with the fact that up to 80% of “idiopathic” POI remains unexplained, suggests that other biologically significant genetic causes of POI are yet to be identified (3).

Most genes implicated in POI relate to cellular and biological processes that span the female reproductive life course. These include sex differentiation, gonadal differentiation, and gametogenesis. Central to gametogenesis is the unique ability of germ cells to switch from mitosis to meiosis to produce haploid gametes. The timing of this “mitosis-meiosis switch” differs between men and women. In males, testicular primordial germ cells (PGCs) undergo mitotic expansion but do not enter meiosis until the onset of spermatogenesis in the postpubertal male. In females, PGCs undergo rapid mitotic proliferation and expansion during fetal life before entering meiosis, with a peak number of cells undergoing meiosis at 16 weeks postconception (wpc). Indeed, meiotic entry and progression are crucial for generating a woman’s lifelong ovarian follicle reserve, and this process may be required to maintain the integrity of the ovary itself (12).

Pathogenic variants within several genes required for meiotic progression have been implicated in the pathogenesis of POI — as examples, *BRCA2*, *MCM8*, *MCM9*, and *STAG3* (13–15). Abnormalities of any meiotic process could theoretically result in the clinical phenotype of POI, yielding many potential candidate genes. Studies in mice have revealed mammalian meiosis to be a complex program with multiple layers of transcriptional regulation, including retinoic acid signaling, bone morphogenetic protein signaling, and expression of key factors required for meiotic entry and progression including DAZL, MSX1, MSX2, and MEIOSIN-STRA8 (16–20). However, mutations implicated in human gonadal insufficiency currently overlap with only a small number of genes known to be involved in the mouse meiotic program. Furthermore, genes expressed specifically during human meiosis have been identified, such as *SOX15* and *SOX17* during PGC specification (21–23). We have to date only a rudimentary knowledge of the human meiotic program, which precludes a full understanding of the pathogenesis of POI. It is likely that key genetic and epigenetic mechanisms underlying human ovary development, and specifically meiosis, have yet to be elucidated, and aberrations within these mechanisms may account for a subset of unexplained POI.

Here, we explore the role of the N6-methyladenosine (m^6^A) reader RNA helicase *YTHDC2* in human ovarian function and meiosis and associate variants within this gene with POI.

## Results

### YTHDC2 variants in women with early-onset POI.

Exome sequencing of a cohort of women with unexplained early-onset POI revealed pathogenic variants in *YTHDC2* in 3 women from 2 families. All 3 patients presented with a severe phenotype of absent puberty and primary amenorrhea and were diagnosed with POI at an early age (Table 1). Extended characterization for the etiology of ovarian insufficiency demonstrated a negative screen for adrenal and ovary autoantibodies, a normal 46,XX karyotype (excluding Turner syndrome), and negative FRAXA premutation analysis (excluding fragile X syndrome). Pelvic imaging revealed no ovaries or very-small-volume ovarian tissue (24). All young women progressed through puberty normally after hormone replacement therapy was commenced.

**Figure 1:**
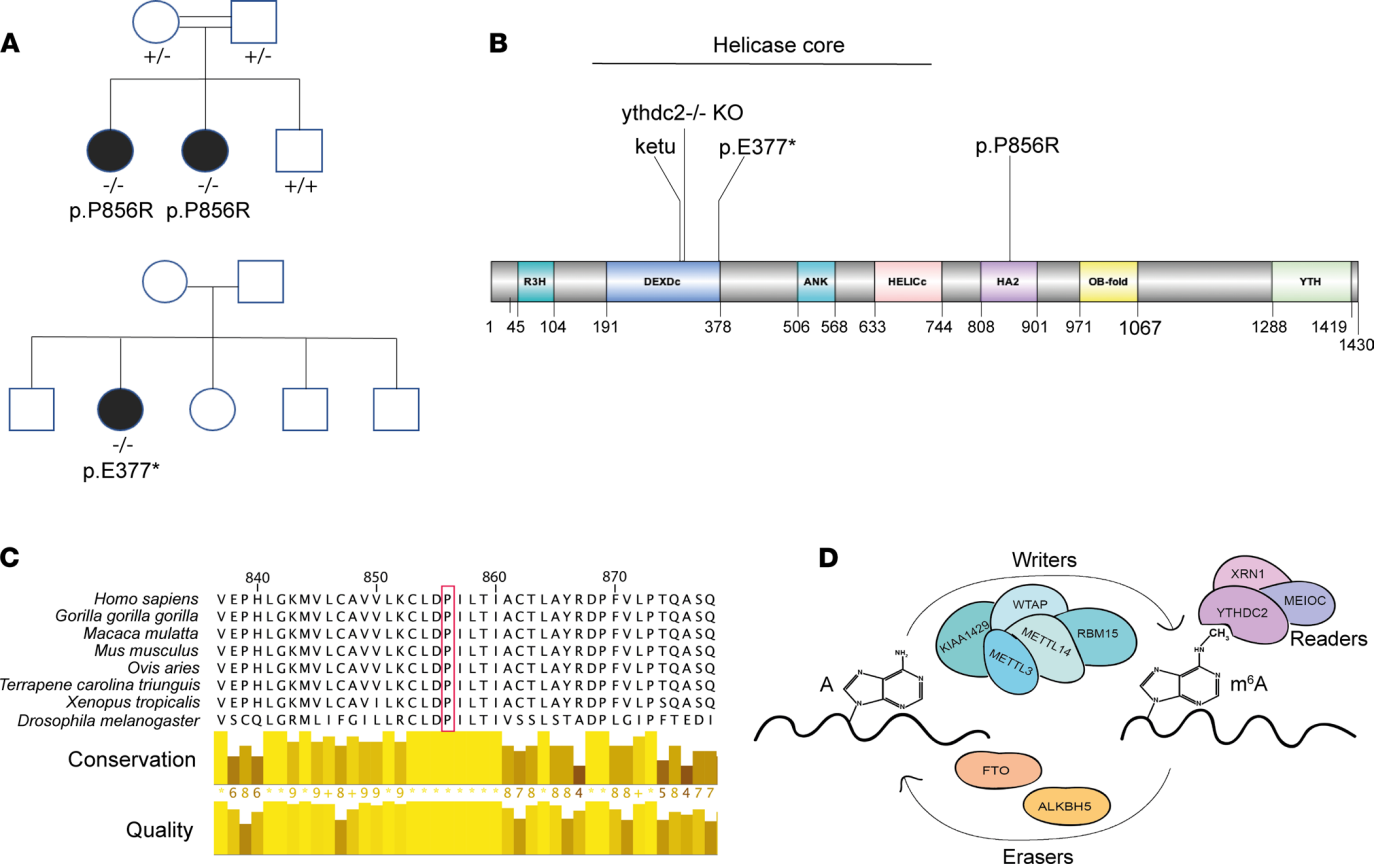
YTHDC2 variants identified. (**A**) Pedigrees of kindred with *YTHDC2* variants (upper panel: p.P856R, lower panel: p.E377*). Solid symbols indicate affected family members. Genotype is indicated underneath tested family members. (**B**) Domains of the human YTHDC2 protein. Domains include an R3H domain; 2 RecA-like domains, RecA1: ATP-binding DEAD-like helicase domain (DExDc) and RecA2: helicase superfamily C-terminal domain (HELICc); 2 ankyrin repeats (506–535 and 539–568) comprising the ankyrin domain (ANK); a helicase-associated domain (HA2), an oligonucleotide/oligosaccharide-binding fold domain (OB-fold), and a YTH (YT521-B homology) m^6^A-dependent RNA binding domain (YTH). The DExDc, ankyrin repeats, and HELICc domains make up the helicase core of this protein. Domain positions (in amino acids) are annotated below the schematic. The position of the 2 pathogenic variants (p.E377*; p.P856R); the *ketu* mouse mutation (mouse: p.H327R; human: p.H312R) (Jain et al. 2017, ref. 26); and another *Ythdc2*^–/–^ knockout mouse (deletion of 50 amino acids within exon 7) (Wojtas et al. 2017, ref. 27) are indicated. (**C**) Amino acid conservancy in the region of YTHDC2 surrounding codon 856. Yellow asterisks represent complete conservation among the species shown. (**D**) The m^6^A methyltransferase complex. The m^6^A modification on mRNA is mediated by “writers” and demethylases (“erasers”). “Readers” recognize the m^6^A modification and allow execution of its functions. One key reader is *YTHDC2*, with its known cofactors *MEIOC* and *XRN*.

In one consanguineous family from eastern Gujarat in India, the 2 affected sisters (patients 1.1 and 1.2) had a homozygous single nucleotide substitution, c.2567C>G, p.P856R, in *YTHDC2* at position chr5:g.113563983C>G (exon 20; RefSeq transcript NM_022828.5, GRCh38) (Figure 1A). This change was the only biallelic nonsynonymous variant that cosegregated with the POI phenotype under an autosomal recessive mode of inheritance in the family, where parents are heterozygous and the affected siblings are homozygous (Figure 1A and Supplemental Figure 1; supplemental material available online with this article; https://doi.org/10.1172/jci.insight.154671DS1). The unaffected brother (WT homozygote) progressed through puberty and had a normal reproductive endocrine profile.

The detected p.P856R variant replaces a basic proline with polar arginine in the helicase-associated (HA2) domain of the YTHDC2 protein (Figure 1B). This results in a protein predicted to be damaging in all in silico models studied (Combined Annotation Dependent Depletion score 27.0) (Supplemental Table 1). The affected amino acid is located within the HA2 domain and is highly conserved in YTHDC2 across different species (Figure 1C), and the variant is not present in public databases (gnomAD v.2.1.1 and v.3.1.2; >400,000 control alleles and >35,000 South Asian alleles) (25).

In a second family, a homozygous single nucleotide substitution, c.1129G>T, resulting in a stop-gain variant (p.E377*) in *YTHDC2* at position chr5:g.113539100G>T (exon 8), was identified in patient 2 (Table 1, Supplemental Table 1, and Figure 1A). Her unaffected parents originate from northern Pakistan. Three brothers and 1 sister were clinically unaffected (not tested). This stop-gain mutation is within the helicase core of the YTHDC2 protein and likely results in a truncated protein (Figure 1B). All *YTHDC2* variants were confirmed by Sanger sequencing (Supplemental Figure 1).

### YTHDC2 is expressed in the human ovary and testis during meiosis.

YTHDC2, a 3′-5′ DExH-box RNA helicase, is believed to be a key player in mammalian gonadal development and germ cell function. Disruption of *Ythdc2* in mice has recently been shown to result in impaired meiosis and an ovarian insufficiency phenotype (*ketu* mouse mutation, ref. 26; and other *Ythdc2*^–/–^ knockout mice, refs. 27, 28). YTHDC2 binds to partner proteins MEIOC and XRN1 (Figure 1D) (27, 29, 30). YTHDC2 is a “reader” of the N6-methyladenosine methyltransferase (m^6^a) complex, an mRNA modification with roles in posttranscriptional gene expression control.

**Figure 2:**
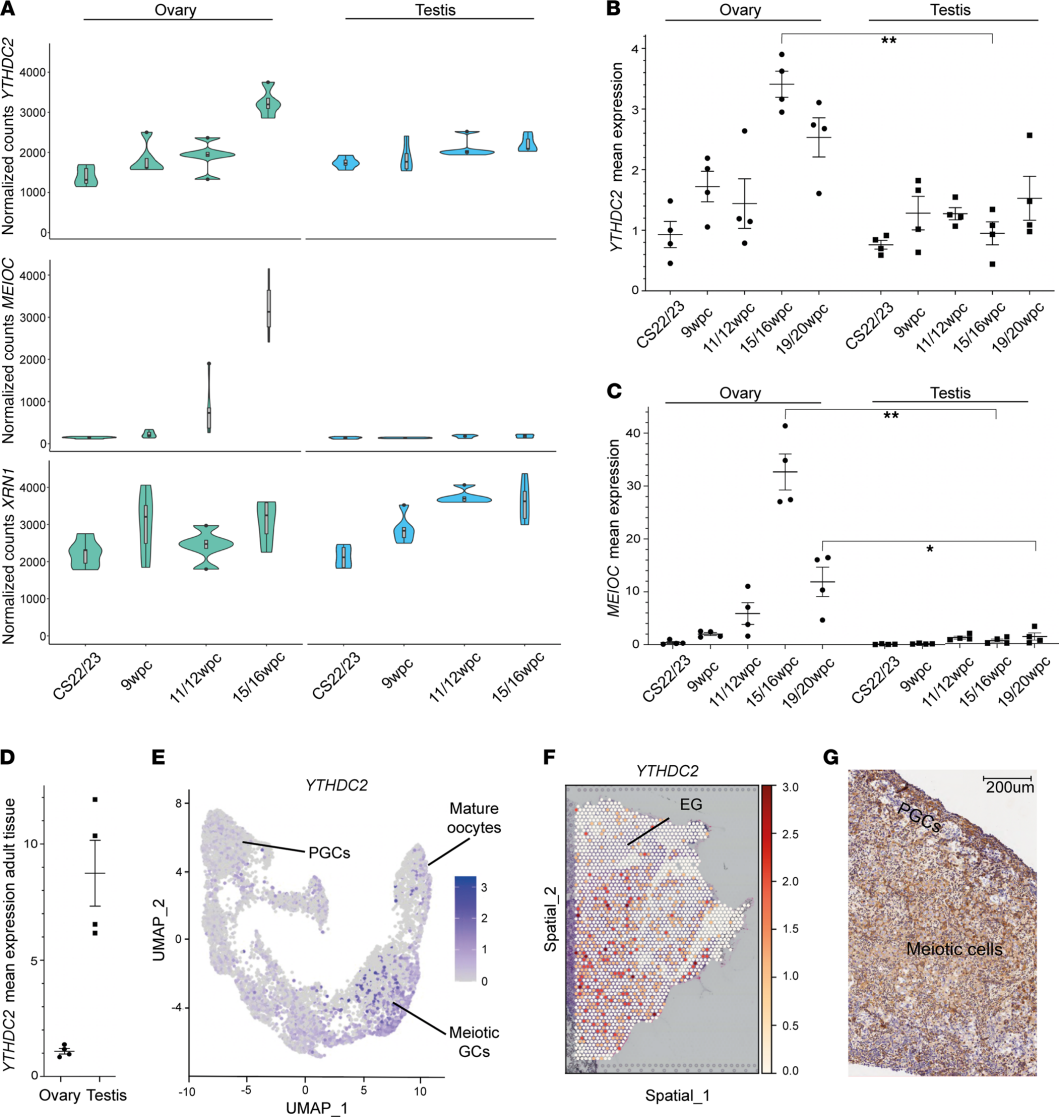
YTHDC2 expression in human fetal gonadal development. (**A**) Violin plots depicting normalized counts for *YTHDC2*, *MEIOC*, and *XRN1* in the developing ovary and testis at 4 developmental stages (4 samples at stages CS22/23, 9 wpc, 11 wpc, 15/16 wpc). (**B**) Quantitative reverse transcriptase PCR (qRT-PCR) expression (log_2_) of *YTHDC2* in developing ovary and testis compared with reference (*ACTB*) and relative to expression in an index CS22 ovary sample. Four fetal ovary and fetal testis samples were included at CS22/CS23, 9 wpc, 11 wpc, 15/16 wpc, and 19/20 wpc. (**C**) qRT-PCR expression (log_2_) of *MEIOC* in developing ovary and testis compared with reference (*ACTB*) and relative to the expression in a CS22 ovary sample. Four fetal ovary and fetal testis samples were included at CS22/CS23, 9 wpc, 11 wpc, 15/16 wpc, and 19/20 wpc. (**D**) qRT-PCR mean expression (log_2_) of *YTHDC2* in 4 adult ovary samples and 4 adult testis samples from individuals without gonadal insufficiency. **B**–**D** represent mean expression of triplicates, and error bars indicate mean ± SEM. qRT-PCR experiments were performed 3 times; representative data from 1 experiment shown. Differences in mean expression between sample groups were assessed using multiple *t* test analysis (*P < 0.05; **P < 0.01); correction for multiple comparisons was performed using Holm-Šídák. CS, Carnegie stage. (**E**) Uniform manifold approximation and projection representation of distinct germ cell clusters (*n* = 8867 germ cells) identified by single-cell RNA sequencing of human fetal gonads between 6 and 21 wpc. Key ovary clusters are annotated. PGCs, primordial germ cells; GCs, germ cells. (**F**) Visium spatial transcriptomic expression of *YTHDC2* in a 17 wpc human fetal ovary. EG, extragonadal. (**G**) Immunohistochemistry of YTHDC2 in a 16 wpc human fetal ovary showing increased staining in the central region containing meiotic cells and decreased staining in primordial germ cells at the periphery in the cortex of the fetal ovary (PGCs).

Given its postulated role in meiosis initiation, we studied expression of *YTHDC2* and genes encoding partner proteins MEIOC and XRN1 at critical stages of human fetal gonad development, including sex differentiation, germ cell expansion, and meiosis onset (CS22/23 [7.5–8 wpc], 9 wpc, 11 wpc, 15/16 wpc, and 19/20 wpc) (Figure 2). Bulk sequencing of RNA from tissues across these stages showed increased *YTHDC2* expression in the late (15/16 wpc) compared with the early (CS22/23) ovary (log_2_ fold change [log_2_FC] 1.14, adjusted *P* [p.adj] < 0.05), consistent with increasing meiosis at that time (Figure 2A). A similar expression pattern of *MEIOC* was also observed when comparing these time points, whereas *XRN1* expression was not increased (Figure 2A). Higher *YTHDC2* (log_2_FC 0.5, p.adj < 0.05) and *MEIOC* (log_2_FC 5.2, p.adj < 0.05) expression was seen in the ovary compared with testis at 15/16 wpc (Figure 2A). This pattern of gene expression was confirmed by qRT-PCR. Using multiple *t* test analysis, *YTHDC2* expression was higher in the 15/16 wpc ovary compared with testis (adjusted *P* = 0.0007, *t* = 8.564, degrees of freedom [df] = 6); *MEIOC* expression was higher in the 15/16 wpc ovary compared with testis (*P* = 0.0004, *t* = 9.376, df = 6) and in the 19/20 wpc ovary compared with testis (*P* = 0.03, *t* = 3.671, df = 6) (Figure 2, B and C). Furthermore, *YTHDC2* expression was higher in the adult testis, where active meiosis occurs during spermatogenesis, compared with the adult ovary, where meiosis is complete (*P* = 0.0109, *t* = 5.665, df = 3) (Figure 2D).

Single-cell RNA sequencing of germ cells (*n* = 8867) from human fetal gonads (ovaries and testes) between 6 wpc and 21 wpc showed the highest expression of *YTHDC2* in meiotic oogonia of the ovary (Figure 2E) (31). Consistent with this, spatial transcriptomic analysis of human fetal ovary at 17 wpc (31), and immunohistochemistry at 16 wpc, revealed expression of *YTHDC2*/YTHDC2 in more central regions of the cortex, which is enriched for meiotic oogonia, compared with PGCs (Figure 2, F and G) (32).

### Detected YTHDC2 variants do not affect RNA stability and splicing in peripheral leukocytes.

**Figure 3:**
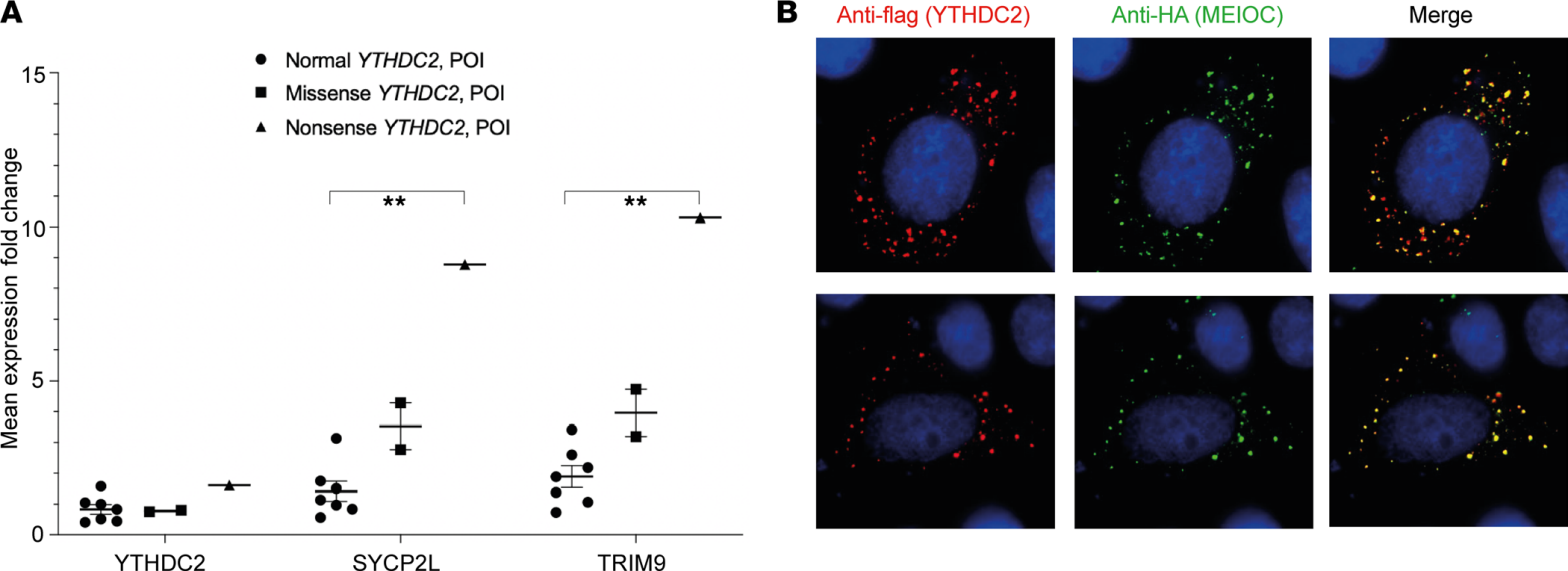
YTHDC2 expression and stability in peripheral leukocytes of patients with YTHDC2 variants compared with controls. (**A**) qRT-PCR mean expression (log_2_) of *YTHDC2*, *SYCP2L*, and *TRIM9* in the peripheral leukocytes of patients with p.P856R (missense) and p.E377* (nonsense) *YTHDC2* variants compared with reference (*ACTB*) and relative to the expression in patients with POI who have no known *YTHDC2* variants. Samples were processed in triplicate and error bars indicate mean ± SEM (**P < 0.01, ANOVA 1 way comparing gene expression between the 3 groups). Experiments were performed 3 times and representative data from 1 experiment are shown. (**B**) Cellular localization studies of either WT or mutant p.P856R YTHDC2 together with MEIOC in HeLa cells. Original magnification, ×100.

Peripheral leukocytes were used as a biological system to investigate the effect of the *YTHDC2* variants on the stability and splicing of the *YTHDC2* transcript, as these cells express *YTHDC2* (qRT-PCR and Genotype-Tissue Expression data; ref. 33) (Supplemental Figure 2A). RNA sequencing of leukocytes from the fresh blood of the 3 patients with *YTHDC2* changes (c.2567C>G, p.P856R; c.1129G>T, p.E377*) and 7 patients with no pathogenic changes in *YTHDC2* revealed *YTHDC2* transcript levels to be similar between the 2 groups (Supplemental Figure 2B). This was confirmed on qRT-PCR (Figure 3A). Furthermore, differential transcript analysis using DEXSeq bioinformatic analysis showed no differences in exon usage or exon splicing between patients and controls (FDR < 10%) (Supplemental Figure 2C). Taken together, these findings suggest that *YTHDC2* variants do not affect RNA stability or splicing of the *YTHDC2* transcript within peripheral leukocytes.

### Global transcriptomic differences between patients with YTHDC2 variants and controls.

We analyzed the peripheral leukocyte RNA-sequencing data set for global transcriptomic differences between patients with *YTHDC2* variants and controls. Principal component analysis (PCA) demonstrated that PC3 (principal component 3; 12.0% of variance) and PC4 (10.4% of variance) separated both groups (Supplemental Figure 2, D and E). Gene expression analysis revealed 13 differentially expressed genes in *YTHDC2* variants compared with controls (absolute log_2_FC > 0.5, p.adj < 0.05) (Supplemental Figure 2F). Genes of interest were validated by qRT-PCR. *TRIM9*, an E3 ubiquitin-protein ligase, and *SYCP2L*, a paralog of meiosis-specific synaptonemal complex protein 2 (SYCP2), were upregulated in patients with *YTHDC2* variants compared with controls (Figure 3A). Using thresholds of log_2_FC > 0.5 and p.adj < 0.05, there were no differentially expressed transposable elements (TEs) when comparing patients with *YTHDC2* variants and controls (TEtranscripts).

### The YTHDC2 missense change p.P856R does not affect the YTHDC2-MEIOC interaction.

We assessed the cellular expression and localization of the pP856R missense variant in an in vitro cell system. Transfection of WT or p.P856R FLAG-tagged YTHDC2 with HA-tagged MEIOC into HeLa cells showed similar cytoplasmic localization (Figure 3B). In most cells, WT or p.P856R YTHDC2 colocalized with MEIOC (Figure 3B). Both WT and p.P856R YTHDC2 associated with MEIOC in co-immunoprecipitation assays (Supplemental Figure 3). These studies suggest that YTHDC2/MEIOC interactions are not significantly affected by the p.P856R variant.

### The YTHDC2 p.P856R variant results in a conformational change of the native protein.

**Figure 4:**
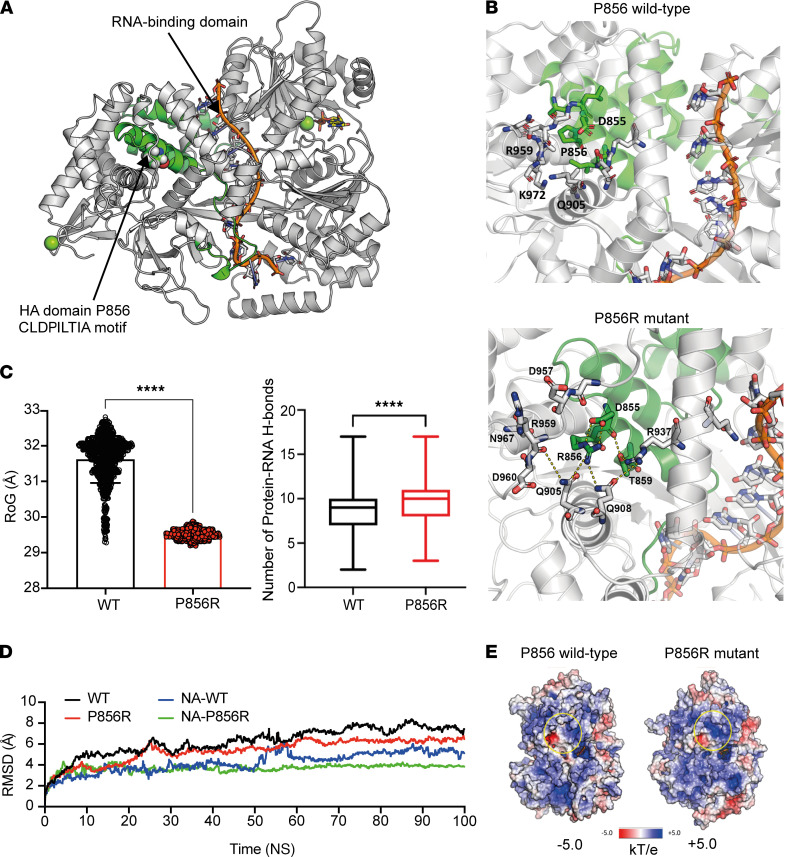
Structural modeling and simulation of human YTHDC2. (**A**) The HA2 domain is shown in green and codon P856 is indicated by an arrow. The RNA-binding domain is shown with the bound RNA in orange. (**B**) P856R can establish strong hydrogen bond interactions with surrounding residues (D855,T859, Q905, Q908, R937, R959, N967), which is predicted to increase stability and to reduce flexibility of the P856R protein. (**C**) The radius of gyration (RoG) indicates that P856R mutant protein is more compact than WT protein (left panel), and there is an increased number of protein-RNA hydrogen bonds in the P856R protein compared with WT (right panel), suggesting the P856R protein to be more stable and less flexible than WT. The box plots depict the minimum and maximum values (whiskers), the upper and lower quartiles, and the median. The length of the box represents the interquartile range. ****P < 0.0001, unpaired *t* test. (**D**) Root-mean square deviation (RMSD) of alpha carbons at the protein and nucleic acid (NA) level for both WT and P856R mutant YTHDC2. Global movement is lower in the P856R mutant protein compared with WT. (**E**) Replacement of proline at position 856 with arginine may also alter the electrostatic charge of YTHDC2.

Next, we characterized the p.P856R YTHDC2 missense change using in silico modeling (Figure 4A). DExH-box helicases bind RNA (ATP-independent) and unwind this duplex RNA via progressive 3′-5′ translocation on single-stranded RNA (ATP-dependent) (34). The biological action of YTHDC2 is likely the product of coordinated RNA binding, RNA unwinding, and RNA helicase enzymatic activity mediated by its heterogeneous domains. The proline residue at 856 of the HA2 domain is not predicted to interact with RNA directly (Figure 4A). However, the p.P856R change results in replacement of a nonpolar proline residue with a larger charged polar arginine, resulting in strong hydrogen bonds between residue R856 and residues Q905 and D855 (intrahelical R-D ion pair) and a higher number of protein-RNA hydrogen bonds (Figure 4, B and C). These changes are predicted to lead a more stable, compact protein with a lower RoG that is less flexible and tolerant for the conformational changes needed for RNA processing (35, 36) (Figure 4, C and D, and Supplemental Figure 5). Predicted changes in surface electrostatic potential could also change protein-protein interactions, although this change is considered of lower importance given the protein size (Figure 4E and Supplemental Figure 5).

### YTHDC2 is associated with the human female meiotic program.

**Figure 5:**
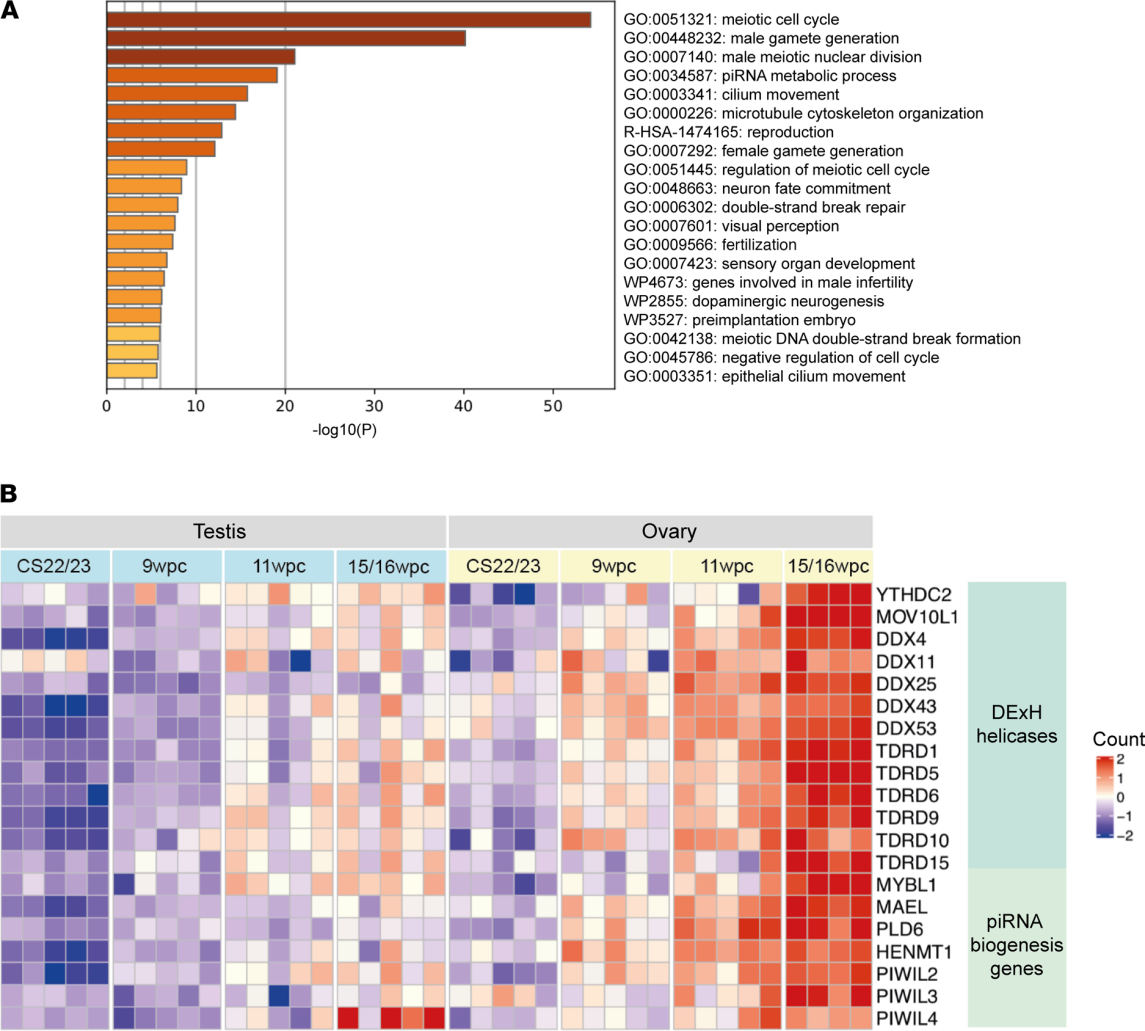
Human YTHDC2 expression during meiosis. (**A**) Pathway enrichment analysis based on genes showing increased expression at 15/16 wpc compared with CS22/23 (log_2_FC > 1, p.adj < 0.05) (cluster/group 1, Supplemental Figure 6). (**B**) Heatmap representing differential gene expression analysis of DExH helicases and piRNA biogenesis genes when comparing ovary 15/16 wpc to testis 15/16 wpc (log_2_FC > 1, p.adj < 0.05). The intensity of gene expression is indicated by a color scale: violet for lowest expression and red for highest expression. Data are expressed as scaled counts.

To visualize groups of genes presenting similar expression patterns, time-series bulk RNA-sequencing data were used to examine the expression of *YTHDC2* within the context of known meiotic markers. Cluster analysis of all ovary samples (CS22/23 vs. 15/16 wpc, log_2_FC >1, p.adj < 0.05) revealed a cosegregation of genes (cluster/group 1) with increasing upregulation from CS22/23 to 15/16 wpc (Supplemental Figure 6A). This cluster included *YTHDC2* as well as known meiosis-specific markers, such as *MEIOC*, *STRA8*, *SPATA22*, *MEIOB*, *DMC1*, *SYCP3*, *HFM1*, and *SYCP1* (e.g., *MEIOC* log_2_FC 5.26, p.adj < 0.05; STRA8 log_2_FC 7.1, p.adj < 0.05) (Supplemental Figure 6, A and B). Pathway enrichment analysis mapped genes within cluster/group 1 to meiosis-specific processes (Figure 5A) and to female and male gonadal insufficiency (Supplemental Figure 6C). Of note, piwi-interacting RNA (piRNA) metabolic processes, cilium movement, and microtubule cytoskeleton organization were highly significant biological components identified (log_10_*P* –17.48). The piRNA metabolic process function contained a distinct subset of genes that related to piRNA metabolism, including known piRNA biogenesis factors such as *PIWIL2-4*, *HENMT1*, *PLD6*, and *MAEL* (37–39). Several RNA DExH/D helicases were also included within this cluster (e.g., *DDX4*, *TDRD9*). Abnormal *Ythdc2* has been associated with dysregulated piRNA metabolism and RNA helicase function and aberrant meiotic progression in mice (26–28). Differential gene expression analysis (ovary 15/16 wpc versus testis 15/16 wpc, log_2_FC >1, p.adj < 0.05) revealed that these RNA helicases and piRNA-associated genes were highly expressed in the fetal ovary at 15/16 wpc, corresponding with when the maximum number of cells are in meiosis (Figure 5B).

## Discussion

Here, we provide evidence that the m^6^A reader *YTHDC2* plays a role in the regulation of meiosis and human gonadal development and, for the first time to our knowledge, show that loss of function in *YTHDC2* is associated with POI.

YTHDC2 is a DExH-box RNA helicase that binds to and unwinds RNA via 3′-5′ translocation on single-stranded RNA (26, 27, 34). YTHDC2 is a known reader of the m^6^a complex that, together with erasers and writers, allows posttranscriptional modification of gene expression via splicing regulation (40), m^6^a-marked transcript degradation (41), and promotion of methylated mRNA expression and translation (41, 42). Animal data suggest that the m^6^a complex plays an important role in the posttranscriptional regulation of mammalian gonadal development. Mice and zebrafish deficient in METTL3 and WTAP (writers) (43, 44), ALKBH5 (eraser) (45), and YTHDC1 and YTHDF2 (readers) (46, 47) demonstrate impaired gametogenesis. Similarly, mice deficient in YTHDC2 are infertile with small or atrophied gonads (26–28, 48, 49). Our data suggest that YTHDC2 is also important for human meiosis, with highest expression of *YTHDC2* and *MEIOC* in the fetal ovary and adult testis at times of maximum sex-specific meiotic activity, localization of *YTHDC2* to meiotic germ cells (oogonia) in the fetal ovary, and an increased expression of *YTHDC2* with several known meiotic markers during stages of peak meiosis.

**Figure 6:**
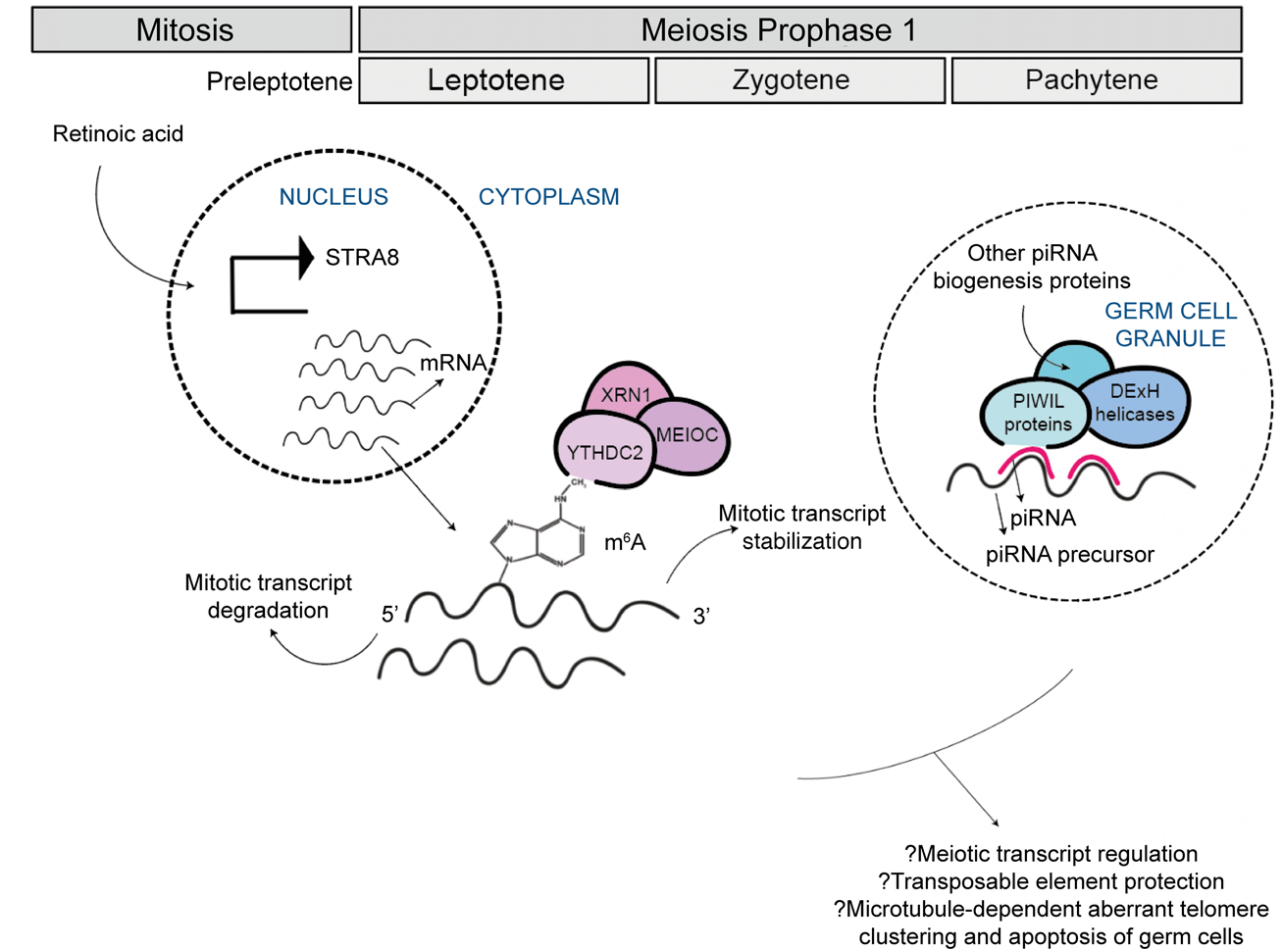
Schematic diagram of hypothesized mechanisms of action of YTHDC2 during human female meiosis. Although the exact role of partners is still incompletely understood, the YTHDC2-MEIOC-XRN1 complex would stabilize meiotic transcripts and/or degrade mitotic transcripts by binding to m^6^A-marked mRNA, promoting a normal mitosis to meiosis transition. At pachytene, YTHDC2 and other DExH helicases may functionally partner with PIWIL proteins within cytoplasmic RNA germ cell granules and may regulate piRNA activity, which is required for normal meiotic timing and progression.

The mitosis-meiosis switch is a key and carefully timed transition during germ cell development. *YTHDC2* is required for the mitosis-meiosis switch in animals. *Ythdc2* and *Meioc* are expressed at the onset of and throughout the extended meiotic phase of prophase 1 in mice (30). Germ cells of *Ythdc2* mutants enter early meiotic prophase but do not complete the mitotic to meiotic transition correctly, with persistent expression of mitotic markers *(cyclin A2*, *cyclin D1*), some expression of early meiotic markers (*Stra8*, *Sycp3*), and no expression of later meiotic markers (*Dmc1*, *Spo11*) (27, 28). As early as preleptotene, germ cells appear to prematurely enter an aberrant metaphase-like state that results in germ cell apoptosis (26, 30, 50). The exact molecular mechanisms underlying the role of *YTHDC2* in the mitosis-meiosis switch have not been fully elucidated, and we did not find impaired YTHDC2-MEIOC interaction in patients with *YTHDC2* variants. However, it is plausible that the YTHDC2-MEIOC complex binds to m^6^a-marked mRNAs to facilitate a correctly timed mitosis to meiosis progression in humans. This is supported by the finding in mice that YTHDC2 and MEIOC pull down mitosis-associated transcripts in WT germ cells, suggesting that the YTHDC2-MEIOC complex downregulates the mitotic cell cycle program (30). Indeed, given what is known about the function of other m^6^a readers, YTHDC2 may both promote the translation efficiency of required meiotic transcripts and degrade unwanted transcripts used earlier in the process — the latter possibly mediated via XRN1 (27) (Figure 6). This may explain the higher expression of peripheral leucocyte expression of *SYCP2L* in patients with *YTHDC2* variants compared with controls. SYCP2L, a synaptonemal complex protein, is an early meiotic marker, and defects in this gene have very recently been reported in association with POI (51). *Ythdc2* mutant mice can express *Sycp2l* (leptotene) but not later meiotic markers (pachytene) (26). Abnormal *YTHDC2* appears to prevent m^6^a-mediated degradation of mitotic and early meiotic markers such as *SYCP2L*, impeding normal meiotic progression and possibly explaining the higher expression of *SYCP2L* in patients with *YTHDC2* variants. A faulty mitotic-meiotic switch represents a severe and early folliculogenesis block and may explain the small-volume, follicle-deplete ovaries seen in the 3 patients with early-onset POI and *YTHDC2* variants.

The YTHDC2 variants identified in the 3 women with early-onset POI likely have different functional consequences. The biallelic stop-gain variant (c.1129G>T, p.E377*) might typically be predicted to result in nonsense-mediated decay; however, we found that this transcript appears to avoid nonsense-mediated decay in patient leukocyte studies and so is more likely to be translated into a protein that is truncated after the amino-terminal DExDc motif of the RNA helicase core. Notably, several *Ythdc2* mouse models involve disruption within or near this highly conserved YTHDC2 helicase core. In addition, we identified a biallelic c.2567C>G variant in 2 sisters that is predicted to replace a basic proline with a polar arginine (p.P856R) in the HA2 domain of the YTHDC2 protein and that does not influence transcript splicing or stability. Investigation of this variant in vitro did not show clear disruption of YTHDC2 expression or YTHDC2/MEIOC colocalization. We hypothesized that the p.P856R variant may induce a conformational change affecting downstream function. The HA2 domain is found in many DExH-box RNA helicases; conserved motifs within the helicase core bind to RNA, promote ATP binding and hydrolysis, and — via their tertiary structure — result in the formation of an RNA-binding tunnel that facilitates efficient substrate loading (52–54). The RNA-binding domains of DExH-box RNA helicases may indirectly regulate helicase activity because their deletion reduces the rate of unwinding activity (54). Deletion of the HA2 domain results in undetectable RNA unwinding, although RNA binding is preserved (54). Confirmational kinetics may therefore play a particular role here, with the HA2 domain acting as a “wedge” to prevent reannealing of the newly separated single-stranded RNAs (54). In silico structural modeling of the WT and P856R mutant YTHDC2 protein predicted that substitution of proline for a charged arginine residue would result in hydrogen bonds that increase the stability of this region of the protein, making it more compact and altering the surface charge. This prediction was supported by simulation models, which showed that the p.P856R protein has reduced flexibility, as indicated by a lower RoG. These changes would impede the conformational flexibility required for functions such as RNA unwinding, as well as possibly changing electrostatic potential, which in turn could disrupt protein-protein interactions. Further approaches such as knock-in animal models and RNA-binding studies would be needed to study this in more detail.

As well as a postulated role in the mitosis-meiosis switch, *Ythdc2* has recently been suggested to have a later role at pachytene of meiosis 1, and this is also suggested by our data (49). A male *Ythdc2* mouse model deficient for YTHDC2 at pachytene demonstrated aberrant microtubule network changes and telomere clustering with germ cell apoptosis (49). Here, we gained further insight into the role of *YTHDC2* in the developing human ovary from cluster and pathway enrichment analyses of differentially expressed genes between early- and late-stage ovaries throughout human prophase 1. A distinct subset of genes within this cluster related to piRNA biogenesis, including *PIWIL2-4*, *HENMT1*, *PLD6*, and *MAEL* (37–39). At pachytene, MYBL1 initiates transcription of piRNA biogenesis genes and piRNA precursors (55). piRNAs then accumulate in cytoplasmic germ cell granules, where they are suggested to protect the integrity of germ cells against TEs and to regulate gene expression by directing PIWI proteins to cleave target meiotic mRNAs at the appropriate times (56, 57). Downstream targets of murine MYBL1 include MILI (PIWIL2), MAEL, TDRD9, MOV10L1, DDX39A, and VASA (DDX4) (55). Consistent with a role in piRNA metabolism, TDRD9- and MOV10L1-deficient mice and VASA- and DDX3-deficient *Drosophila* demonstrate derepressed TEs, abnormal piRNA profiles, and meiotic arrest within germ cells (39, 58–60). Similarly, *Ythdc2-*deficient mice show downregulation of 1 piRNA precursor, impaired interaction with MIWI (murine PIWIL1) and MSY2 (murine YBX2), and downregulation of *Miwi* within germ cell granules (28, 49). In humans, *TDRD9* pathogenic variants are associated with male factor infertility (61), *DHX37* variants with 46,XY gonadal dysgenesis (62), and here, *YTHDC2* variants with POI. Most of these above-described genes are either piRNA biogenesis or DExH/D helicase genes; furthermore, the 3 latter genes are paralogs and share both a helicase core and HA2 domain. We speculate that DExH/D helicases have a role at pachytene, supported by our observation of perinuclear YTHDC2 staining and upregulation of DExH/D helicases, *PIWIL* genes, and other piRNA biogenesis genes at meiosis.

We did not find differential TE expression in the peripheral leukocytes of patients with *YTHDC2* variants compared with controls, suggesting that any potential role of *YTHDC2* in piRNA-mediated TE regulation is meiosis specific. However, in addition to dysregulated TE expression, PIWI knockdown in a *Drosophila* ovarian follicle cell line was found to indirectly regulate a small subset of protein-coding genes — including *TRIM9*, which was 3-fold upregulated in the PIWI knockdown model (63). We show that *TRIM9* was more highly expressed in the peripheral leukocytes of patients with *YTHDC2* variants, intimating that piRNA-mediated TE disruption during meiosis may result in enduring downstream genomic changes. Furthermore, a functioning piRNA pathway protects against TEs, and this protection is required for normal microtubule organization during gonadal development in *Drosophila* (64, 65). We noted dual clustering of piRNA and microtubule processes at human meiosis and additionally noted the abnormal microtubule function in murine germ cells deficient in *Ythdc2* at pachytene (49). Taken together, we speculate that *YTHDC2*, both an m^6^A-reader and a DExH helicase, may have dual meiotic roles in humans: m^6^A-mediated processing of mRNA transcripts in the mitosis-meiosis switch of early prophase and a later role in piRNA processing within germ cell granules at pachytene (Figure 6) (59). We suggest that human *YTHDC2* variants result in several meiotic abnormalities similar to *Ythdc2* mutant mice, including persistence of the mitotic program, a dysregulated transcriptomic and epitranscriptomic profile, microtubule-driven aberrant telomere clustering at pachytene, and apoptosis of faulty germ cells.

POI is a condition with high genetic heterogeneity that can pose challenges for clinicians tasked with providing tailored counseling to patients with this diagnosis. A greater understanding of the genetic mechanisms underlying POI has permitted more individualized diagnoses and advice on fertility preservation options and familial risk. Female meiosis and gonadal development require a series of carefully timed biological processes, such as meiosis initiation, synaptonemal complex formation, and homologous recombination. For the most part, POI-associated genetic variants have mapped to these processes in a relatively linear manner. Our association of *YTHDC2* with human meiosis and POI highlights the emerging importance of epitranscriptomic regulation to these processes. It also requires that we widen the network of POI candidate genes to include those that function within posttranscriptional networks, such as m^6^A readers, RNA helicases, and piRNA biogenesis genes. Exploring the posttranscriptional regulation of gonadal development presents challenges as standard mRNA-sequencing techniques are not designed to detect all important signals from non-mRNA species, which is a limitation of our data. This issueis compounded by the inherent difficulty of interrogating a biological process occurring during fetal life within an inaccessible tissue, such as the ovary that atrophies by adult life. Using patient-derived material as surrogate experimental systems, such as used here, addresses these limitations to a degree. However, further studies using epitranscriptomic techniques such as m^6^A-Seq, ATAC-Seq, and small RNA sequencing will be required to fully interrogate the complex transcriptional and posttranscriptional landscape governing female meiosis and to better understand the genetic and epigenetic mechanisms underlying a clinical diagnosis of POI.

## Methods

### Participants.

The affected women in this study were recruited as part of the Reproductive Life Course Study at University College London Hospital, London. Those with a known cause of ovarian dysfunction (e.g., abnormal karyotype, iatrogenic POI) were excluded.

### Genetic analysis.

Exome sequencing was performed using a customized Exome CG enrichment panel (Nonacus) followed by paired-end sequencing on a NovaSeq 6000 (S4, 2 × 151 bp, Illumina) or the BGIseq-500 platform (Beijing Genomics Institute). Reads were aligned against the human reference genome sequence (National Center for Biotechnology Information [NCBI] GRCh38) using the Burrows-Wheeler Aligner (66). Platypus software (v0.8.1) was used for variant calling using standard parameters (67). Multiple nucleotide polymorphisms were normalized and decomposed using vcflib (v1.0). Variant filtering was performed using Ingenuity Variant Analysis software (Qiagen) (Supplemental Figure 1). Variants were kept if they fulfilled the following criteria: read depth ≥ 5, maximum population frequency ≤ 1% (minor allele frequency < 0.01) (gnomAD), and segregation with an autosomal recessive model in the family, where parents are heterozygous for a variant and affected individuals are homozygous. Synonymous changes were excluded, unless they were associated with splice site loss or up to 7 bases into an intron or predicted to affect splicing using MaxEntScan. Potential changes in *YTHDC2* were confirmed using Sanger sequencing (Supplemental Table 2) and compared to gnomAD (v2.1.1 and v3.1.2, https://gnomad.broadinstitute.org, accessed December 2021) (68).

### In silico analysis.

The predicted consequences of *YTHDC2* variants were studied in silico using SIFT (https://sift.bii.a-star.edu.sg), PROVEAN (http://provean.jcvi.org/index.php), and PolyPhen2 (http://genetics.bwh.harvard.edu/pph2/). Conservancy mapping was undertaken using UniProt (https://www.uniprot.org). Protein domain illustration was performed using DOG 1.0 (Illustrator of Protein Domain Structures) (69). Domain annotations were obtained from the PFAM and SMART databases (70, 71).

### RNA expression in human fetal gonad development.

A total of 5 ovaries, 5 testes, and two 46,XX control tissues were included at each of 4 developmental stages: CS22/CS23 (7.5–8 wpc), 9 wpc, 11 wpc, and 15/16 wpc (Supplemental Table 3). Samples were obtained from the Human Developmental Biology Resource (HDBR) (http://www.hdbr.org). RNA was extracted using the AllPrep DNA/RNA Mini Kit (Qiagen). Libraries were prepared using the KAPA RNA HyperPrep Kit and sequenced on an Illumina HiSeq 4000 sequencer at a minimum of 25 million paired-end reads (75 bp) per sample. Reads underwent quality control (FastQC, Babraham Bioinformatics) and were aligned against the human reference genome sequence (NCBI, GRCh38) with STAR aligner (v2.5.2a) (72). featureCounts (Subread package, v2.0.2), DESeq2 (v1.28.1), and degPatterns (DEGreport package, v1.24.1) were used for gene expression quantification, differential gene expression, and detection of expression patterns, respectively (73–75). The Benjamini-Hochberg approach was used to adjust for multiple testing with cutoff of adjusted *P* values of 0.05 (76). Metascape was used for functional enrichment (77). Data are deposited in BioStudies under the accession number S-BSST693.

### qRT-PCR.

*YTHDC2* and *MEIOC* expression were investigated by qRT-PCR using 4 ovaries and 4 testes at each of 5 developmental stages: CS22/CS23 (7.5–8 wpc), 9 wpc, 11 wpc, 15/16 wpc, and 19/20 wpc (HDBR). Four adult ovary (catalog CR560293, CR560115, CR560119, CR560036, Origene) and 4 adult testis (catalog CR561981, CR562064, CR560016, CR562064, Origene) were also included. RNA was extracted as described previously and quantified using a NanoDrop 1000 spectrophotometer (Thermo Fisher Scientific) and reverse-transcribed using the SuperScript III Reverse Transcriptase kit (Thermo Fisher Scientific). qRT-PCR was performed using TaqMan Fast Advanced MasterMix (Applied Biosystems) and TaqMan assays (*YTHDC2:* Hs00403320_m1; *MEIOC*: Hs00403320_m1; Applied Biosystems) on an ABI StepOne Plus System (Applied Biosystems). The relative expressions of *YTHDC2* and *MEIOC* were calculated using the comparative Ct method and *ACTB* (Hs03023943_g1) as a housekeeping gene. Experiments were performed in triplicate on 3 occasions and representative data from 1 experiment are shown. Data are expressed as mean ± SEM.

### Single-cell RNA sequencing and spatial transcriptomics.

Expression of *YTHDC2* during human fetal ovary development was retrieved from single-cell RNA sequencing and spatial transcriptomic data sets from a previous study (31).

### Immunohistochemistry.

A human fetal ovary (16 wpc) was obtained from the HDBR and fixed in 4% paraformaldehyde before being processed, embedded, and sectioned for histology analysis including immunohistochemistry (IHC). The sections for IHC were produced at 4 μm thickness, and staining occurred on Leica Bond-max automated platform (Leica Biosystems). On the Bond-max, the sections underwent antigen retrieval to unmask the epitope (Heat Induced Epitope Retrieval protocol 1 for 20 minutes, pH 6, Bond-max protocol F). Endogenous activity was blocked with peroxidase with bond polymer refine kit (catalog DS9800) followed by incubation with rabbit polyclonal YTHDC2 primary antibody (MilliporeSigma, Merck KGaA, HPA037364, 1:200) for 1 hour. Then, the postprimary was applied to the sections (Bond polymer refine kit; catalog DS9800) and HRP-labeled polymer (Bond polymer refine kit; catalog DS9800). DAB (Bond polymer refine kit; catalog DS9800) chromogen solution was added to the sections to precipitate the locus of antigen-antibody interactions. Next, the sections were counterstained with hematoxylin (Bond polymer refine kit; catalog DS9800), washed in deionized water, dehydrated in graded alcohols, cleared in 2 xylene changes, and mounted for light microscopy. The stained slides were imaged on the Aperio CS2 Scanner (Leica Biosystems, catalog 5872) at 40× objective, and analysis was supported by QuPath (v.0.2.3) and Leica ImageScope (Leica Biosystems) software.

### RNA profiling of peripheral leukocytes.

RNA sequencing was performed on leukocytes obtained from the 3 patients with *YTHDC2* variants and from 7 female “controls” with POI of no known cause. RNA was extracted using the PAXgene Blood RNA kit (Qiagen). Libraries were sequenced with the NovaSeq 4000 (SP, 2 × 151 bp, Illumina). Reads underwent quality control (FastQC, Babraham Bioinformatics) and were aligned against the human reference genome sequence (NCBI, GRCh38) (STAR 2.5.2a) (72). Fastp, DESeq2, DEXSeq, and TEtranscripts were used for preprocessing, differential gene expression analysis, differential exon usage/splicing, and differential transposable element expression, respectively (74, 78–80). Downstream bioinformatic analysis was undertaken as described for fetal samples. Differentially expressed genes of interest were verified using qRT-PCR (*TRIM9:* Hs00364838_m1; *SYCP2L*: Hs00293769_m1; Applied Biosystems).

### Plasmid construction.

Plasmids containing WT or mutated YTHDC2 (P856R) were constructed. Primer sequences are provided in Supplemental Table 2 (all MilliporeSigma). All PCRs were performed using Phusion DNA polymerase (New England Biolabs). All constructs were Sanger sequenced to verify correct insertion of variants and tag epitopes. To build the pcDNA3.1-HsYTHDC2-1-1430-Flag plasmid (SC0827 plasmid), the WT HsYTHDC2-1-1430 gene sequence was first amplified from a plasmid provided by H. Sahara (Azabu University School of Veterinary Medicine, Fuchinobe, Japan), then subcloned into a pcDNA3.1(-) plasmid using NheI and XhoI restriction sites, with a Flag-tag–encoding sequence added to the C-terminus by PCR. The P856R mutation was introduced by rolling circle amplification to produce pcDNA3.1-HsYTHDC2-1-1430-P856R-Flag (SC0828 plasmid). The human cDNA sequence of *MEIOC* was obtained using nested PCR with RNA extracted from 12 and 13 wpc human fetal ovaries following reverse transcription. BamH1 and EcoR1 restriction sites were introduced at the 5′ and 3′ ends of the *MEIOC* sequence for ligation into a pkH3 plasmid containing an HA-tag.

### Subcellular localization of YTHDC2 and MEIOC.

HeLa cells (ATCC) were cultivated in Dulbecco’s modified Eagle medium supplemented with 15% FBS (Gibco) in a 5% CO_2_ humidified incubator at 37°C. Plasmid transfection was performed using Lipofectamine 2000 (Life Technologies) according to the manufacturer’s instructions. HeLa cells were seeded on 170 μm coverslips (Marienfeld) 24 hours before transfection. After 48 hours of transfection, cells were washed twice with PBS with 500 μM MgCl_2_ and 500 μM CaCl_2_ (PBS-S) and fixed using 4% paraformaldehyde. Cells were permeabilized in PBS-S, placed in 0.5% Triton for 10 minutes. and incubated in blocking buffer (PBS-S, BSA 5%, Tween 0.1%) for 30 minutes at room temperature. Coverslips were incubated with primary antibodies (mouse, anti-Flag [clone 9A3], 1:200, Cell Signaling Technology; and rabbit, anti-HA [catalog ab9110], 1:500, Abcam) overnight in blocking solution at 4°C. Cells were then incubated with appropriate fluorescent dye–coupled secondary antibodies (Alexa Fluor AB_2340854 and AB_2313584, Jackson ImmunoResearch) for 1 hour at 37°C in blocking solution, stained for 5 minutes with DAPI, and washed once with PBS-S. Coverslips were mounted on glass slides using ProLong Gold antifade reagent (Thermo Fisher Scientific). Imaging was performed using an AX70 epi-fluorescence microscope (Olympus) equipped with a CoolSNAP MYO charge-coupled device camera (Teledyne Photometrics).

### Co-immunoprecipitation of YTHDC2 and MEIOC.

Human embryonic kidney cells (HEK-293, ATCC) were grown in Dulbecco’s modified Eagle medium supplemented with 10% fetal bovine serum (Gibco) and transfected with plasmids coding for WT or mutant YTHDC2 and MEIOC. HEK-293 cells were lysed using 1× cell lysis buffer (Cell Signaling Technology) supplemented with complete protease inhibitor (Roche) and 10 μM MG-132 (MilliporeSigma) and subjected to medium sonication for 10 minutes (30 seconds on, 30 seconds off) followed by centrifugation at 16,200*g* for 10 minutes at 4°C. Protein extracts were diluted 4 times in binding buffer (NaCl 150 mM, Tris-HCl pH 7.5 20 mM, EDTA 1 mM, glycerol 10%, protease inhibitor 1×, BSA 0.1%). When required, RNase A (Thermo Fisher Scientific) (100 μg/mL) treatment was performed. A total of 600 μL of protein extract and 25 μL of anti-FLAG M2 magnetic beads (MilliporeSigma) were used for each immunoprecipitation. Protein/bead mixtures were incubated overnight at 4°C on a rotating wheel. Beads were washed 4 times with wash buffer (NaCl 150 mM, Tris-HCl pH 7.5 20 mM, EDTA 1 mM, glycerol 10%, protease inhibitor 1×, Tween 0.05%), and immunoprecipitated proteins were eluted in Laemmli buffer supplemented with 0.5% β-mercaptoethanol. Western blotting was performed as previously described (81) using antibodies directed against Flag- (chicken, ab1170, 1:500, Abcam) and HA-tag (rabbit, ab9110, 1:500, Abcam).

### Molecular modeling and simulations of WT and p.P856R variant YTHDC2.

The human YTHDC2 protein sequence was retrieved from the UniProt database (UniProt Q9H6S0) (48). Sequence searches were conducted to identify conserved domain signatures using PFAM (37). Blastp against the PDB database (82) identified the structures of the related RNA helicase proteins MLE (PDB 5AOR) and DHX36 (PDB 5VHE) as potential templates for modeling YTHDC2 and variants (Supplemental Figure 4) (52, 83). Residues 181–421 to 575–1171 of YTHDC2 were finally considered for model generation. We first constructed a model of YTHDC2 based on DHX36, including the nucleic acid and cofactors (ADP + 2 Mg^2+^ atoms). A second model based on MLE without cofactors was also constructed. Finally, we averaged the previously obtained models, by generating a set of 100 models using Modeller v9.25 (84). The best model according to Modeller’s PDF and DOPE scores was selected and verified by Structural Analysis and Verification Server to evaluate its stereo-chemical quality (85, 86). With this approach, structural representations of WT and p.P856R variant YTHDC2 in complex with nucleic acid and cofactors were obtained.

For molecular dynamics (MD) simulations, WT and P856R mutant were solvated and ionized using CHARMM-GUI web server interface (87). The proteins were embedded in a water box of 120 × 120 × 120 Å and the system charges equilibrated with Na^+^ and Cl^–^ atoms up to a concentration of 0.15 M NaCl. The final systems contained approximately 155,000 atoms. All MD simulations were performed using the NAMD v2.14 package (88). The simulation systems were first relaxed with 10,000 steps of minimization followed by gradual heating from 0 to 310°K by running short MD simulations of 500 steps each cycle using the NVT ensemble. The simulation was switched to NPT conditions and was further equilibrated for 2.5 ns while constraining the protein backbone with an initial force constant of 10 kcal/(mol·Å^2^) and gradually decreasing to 8, 6, 4, 2, 1, 0.5, 0.05 kcal/(mol·Å^2^) every 250 ps of MD simulation. Finally, the systems were run without any constraints for 100 ns. Protein, cofactor, and ion atom types and parameters were described by the CHARMM36 force field (89). The van der Waals interactions were calculated applying a cutoff distance of 12 Å and switching the potential from 10 Å. A time step of 2 fs was used in the production phase, and Particle Mesh Ewald was employed for the treatment of long-range electrostatic interactions. The temperature was maintained at 310°K using Langevin dynamics. The Nose-Hoover Langevin piston method was used to control the target pressure (1 atm) with the LangevinPistonPeriod set to 200 fs and LangevinPistonDecay set to 50 fs. The last 100 ns of each simulation was extracted for trajectory analysis using Visual Molecular Dynamics v1.93 software (90). The effect of the arginine substitution at residue 856 was explored by measuring the RMSD of Ca atoms and root-mean square-fluctuation of individual residues. We also measured the RoG, solvent accessible surface area, and hydrogen bond interactions established between protein and nucleic acid as well as by residue R856 through simulation. The differences in electrostatic potential of surface electrostatic potential were calculated using the APBS plugin within PyMOL v2.5 (Schröedinger Inc.) and represented as color spectrum within 5 KT/e units.

### Data availability.

We do not have patient consent to release raw next-generation sequencing data. The data that support the findings of this study are available on request from the corresponding author (SMMB). RNA-sequencing data that support the findings of this study have been deposited in BioStudies under the accession number S-BSST693 at https://www.ebi.ac.uk/biostudies/

### Statistics.

Statistical analyses were performed using GraphPad Prism v9.1.1 (GraphPad Software) and are described in the relevant sections. Student’s *t* tests (2 tailed) were used for statistical testing. Correction for multiple comparisons was performed using the Holm-Šídák method. A *P* value of less than 0.05 was considered significant.

### Study approval.

The affected women recruited to this study provided written informed consent as part of the Reproductive Life Course Study at University College London Hospitals (ethical approval: NRES Committee London-Chelsea [16LO0682]). Human fetal samples were obtained with ethical approval (REC reference 18/LO/0822; 18/NE/0290) and written informed consent from the HDBR (http://www.hdbr.org).

## Author contributions

SMMB, IDV, and PLQS wrote and edited the manuscript. SMMB, IDV, PN, and TB performed RNA-sequencing experiments and analyzed RNA-sequencing data. SMMB, PLQS, TB, DK, LAO, JPS, AG, and MI undertook DNA extraction and exome capture and obtained and/or analyzed exome-sequencing data. CFL performed protein modeling and analysis. FB, MTD, CFL, and DK contributed to discussions and/or data analysis. SMMB, CEB, MTD, and GSC provided clinical data. LB and GL performed cell line expression, localization, and co-immunoprecipitation experiments and provided expert input. LGA and RVT contributed with analysis of single-cell RNA-sequencing and spatial transcriptomic data. BC and NM were involved with tissue selection and dissection (HDBR). OKO and TM performed histopathology experiments. GL, JCA, and GSC supervised the studies and contributed to the writing and editing of the manuscript.

## Supplementary Material

Supplemental data

## Figures and Tables

**Table 1:**
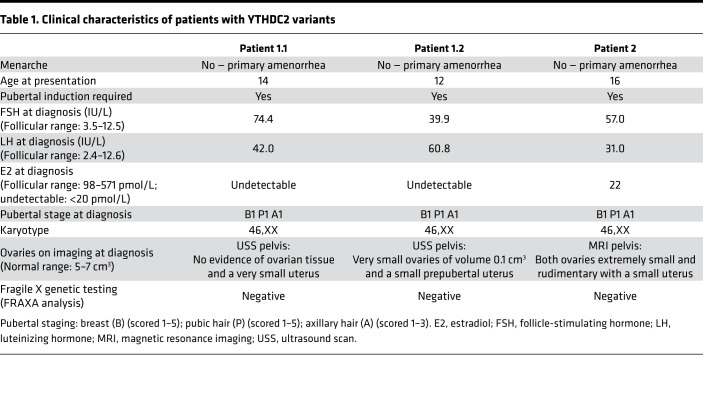
Clinical characteristics of patients with YTHDC2 variants

## Version 1 02/08/2022

In-Press Preview

## Version 2 03/08/2022

Electronic publication
