# Supplementary material for: Pathogenic variants in the human m6A reader YTHDC2 are associated with primary ovarian insufficiency
Source: JCI Insight. 2022 Mar 8;7(5):e154671. doi: 10.1172/jci.insight.154671 (PMC8983136; doi:10.1172/jci.insight.154671)
Supplement: Supplemental data [file jciinsight-7-154671-s150.pdf]

Supplementary Information

Pathogenic variants in the human m^{6}A reader *YTHDC2* are associated with primary ovarian insufficiency

Sinéad M. McGlacken-Byrne[1,2,3*], Ignacio Del Valle[1], Polona Le Quesne Stabej[4,5], Laura Bellutti[6], Luz Garcia-Alonso[7], Louise Ocaka[4], Miho Ishida[1], Jenifer P. Suntharalingham[1], Andrey Gagunashvili[4], Olumide K. Ogunbiyi[8, 9], Talisa Mistry[8, 9], Federica Buonocore[1], GOSgene[4], Berta Crespo[9], Nadjeda Moreno[9], Paola Niola[10], Tony Brooks[10], Caroline E. Brain[2], Mehul T. Dattani[1,2], Daniel Kelberman[4], Roser Vento-Tormo[7], Carlos F. Lagos[11], Gabriel Livera[6], Gerard S. Conway[3], John C. Achermann[1]

[1] Genetics and Genomic Medicine, UCL Great Ormond Street Institute of Child Health, University College London, London WC1N 1EH, United Kingdom

[2] Department of Paediatric Endocrinology, Great Ormond Street Hospital, London WC1N 3JH, United Kingdom

[3] Institute for Women's Health, University College London, London WC1E 6AU, United Kingdom

[4] GOSgene, Genetics and Genomic Medicine, UCL Great Ormond Street Institute of Child Health, University College London, London WC1N 1EH, United Kingdom

[5] Department of Molecular Medicine and Pathology, University of Auckland, Auckland 1142, New Zealand

[6] Laboratory of Development of the Gonads, UMR E008 Genetic Stability Stem Cells and Radiations, Université de Paris, Université Paris Saclay, CEA, F-92265, Fontenay aux Roses, France

[7] Wellcome Sanger Institute, Wellcome Genome Campus, Hinxton, Cambridge CB10 1SA, United Kingdom

[8] Department of Histopathology, Great Ormond Street Hospital for Children NHS Foundation Trust, London WC1N 3JH, UK

[9] Developmental Biology and Cancer, UCL Great Ormond Street Institute of Child Health, University College London, London WC1N 1EH, United Kingdom

[10] UCL Genomics, Zayed Centre for Research, University College London, London WC1N 1DZ, United Kingdom

[11] Chemical Biology & Drug Discovery Lab, Escuela de Química y Farmacia, Facultad de Medicina y Ciencia, Universidad San Sebastián, Lota 2465, Providencia 7510157, Santiago, Chile

GOSgene consortium details (in addition to listed authors affiliated with GOSgene):

Chela James[2]

Hywel J. Williams[2]

Chiara Bacchelli[2]

[2] GOSgene, Genetics and Genomic Medicine, UCL Great Ormond Street Institute of Child Health, University College London, London WC1N 1EH, United Kingdom

Supplementary Table 1: *YTHDC2* variants and predictions of pathogenicity

	**Patient 1.1**	**Patient 1.2**	**Patient 2**
**Gene symbol**	*YTHDC2*		
**Full gene name**	YTH domain containing 2		
**OMIM clinical phenotype**	None		
**Position**	chr5:113563983	chr5:113563983	chr5:113539100
**Region**	Exonic	Exonic	Exonic
RefSeq transcript NM_022828.5	NM_022828.5	NM_022828.5	NM_022828.5
**Exon involved**	Exon 20 of 30 exons	Exon 20 of 30 exons	Exon 8 of 30 exons
**cDNA variant**	c.2567C>G	c.2567C>G	c.1129G>T
**Protein variant**	p.P856R (proline → arginine)	p.P856R (proline → arginine)	p.E377* (glutamic acid → null)
**Translation impact**	Missense	Missense	Stop gain
**Genotype**	Homozygous	Homozygous	Homozygous
**Read depth**	89	92	14
**Allele fraction**	100	97.95	100
**SIFT function prediction**	Damaging	Damaging	
**Poly-Phen2 function prediction**	Probably Damaging	Probably Damaging	N/A
**CADD score**	27.0	27.0	44.0
**PROVEAN score**	Deleterious	Deleterious	N/A
**Mutation Taster**	-	-	Disease-causing
**Allele frequency in gnomAD**	0	0	0
**Presence in dbSNP**	Not present	Not present	Not present
**Gene constraint from gnomAD** – **Missense (observed *vs* expected, +/- 90% CI)**	Z score: 0.33<br>Expected SNVs: 736.8<br>Observed SNVs: 712<br>o/e 0.97 (CI 0.91-1.03)		
**Gene constraint from gnomAD** – **Loss of function (observed *vs* expected, +/- 90% CI)**	pLI score: 1.0 (extremely intolerant to loss of function)<br>Expected SNVs: 80.1<br>Observed SNVs: 5<br>o/e 0.06 (CI 0.03-0.13)		

Note: Although constraint for missense changes was not observed, detailed analysis of gnomAD v2.1.1 showed that only five individuals out of >125,000 subjects had predicted damaging/deleterious non-synonymous missense changes in *YTHDC2*.

Supplementary Table 2: Primers used for Sanger sequencing of patient *YTHDC2* variants and for plasmid construction

**Primer name**	**Sequence (5' → 3')**
Primers used for Sanger sequencing to confirm YTHDC2 *variants in affected women*	
p.P856R YTHDC2_F	GTCATGTATGAAACAGTAGATAT
p.P856R YTHDC2_R	AGAAACGTGTTTACCCAGCAACG
p.E377* YTHDC2_F	AAACCCAAGCACACAAACAG
p.E377* YTHDC2_R	GTGTCCTTGTACCTCCACAAT
Primers used for plasmid construction	
HsYTHDC2_F	CTATAGGGAGACCCAAGCTGGCTAGCATGTCCAGGCCGAGC
HsYTHDC2_R	CTTGTCGTCATCGTCTTTGTAGTCGCCTCCATCAGTTGTGTTTTTTTCTCCC
HsYTHDC2_R2	CACAGTGGCGGCCGCTCGAGTCACTTGTCGTCATCGTCTTTGTAG
HsYTHDC2_F_mut2567C>G	GGACCGCATCCTTACAATTGCTTGCACACTAGC
HsYTHDC2_R_mut2567C>G	TAAGGATGCGGTCCAGACACTTTAAAACAACAGCACAC
hMEIOC_UTR5'_F	TGTGCCTAGTCCAGGAGAG
hMEIOC_UTR3'_R	TCTTCTTTCTGCTGGCATTTCC
hMEIOC_FW_CGBamHI_F	CGGGATCCGAGGTGAGACGCGGAGAC
hMEIOC_RV_CGEcoRI_R	CGGAATTCTTAATGTTTGTTTGTTTCACCTCTC

Supplementary Table 3: Fetal tissue samples included in the time series bulk RNA sequencing experiment (n=47).

**Tissue**	**Stage**	**Sample number**
Ovary	CS22/23	5
Ovary	9wpc	5
Ovary	11/12wpc	5
Ovary	15/16wpc	4
Testis	CS22/23	5
Testis	9wpc	5
Testis	11/12wpc	5
Testis	15/16wpc	5
46,XX kidney	CS22	1
46,XX skin	CS22	1
46,XX muscle	9wpc	1
46,XX spleen	9wpc	1
46,XX stomach	11wpc	1
46,XX lung	11wpc	1
46,XX pancreas	15wpc	1
46,XX skin	15wpc	1

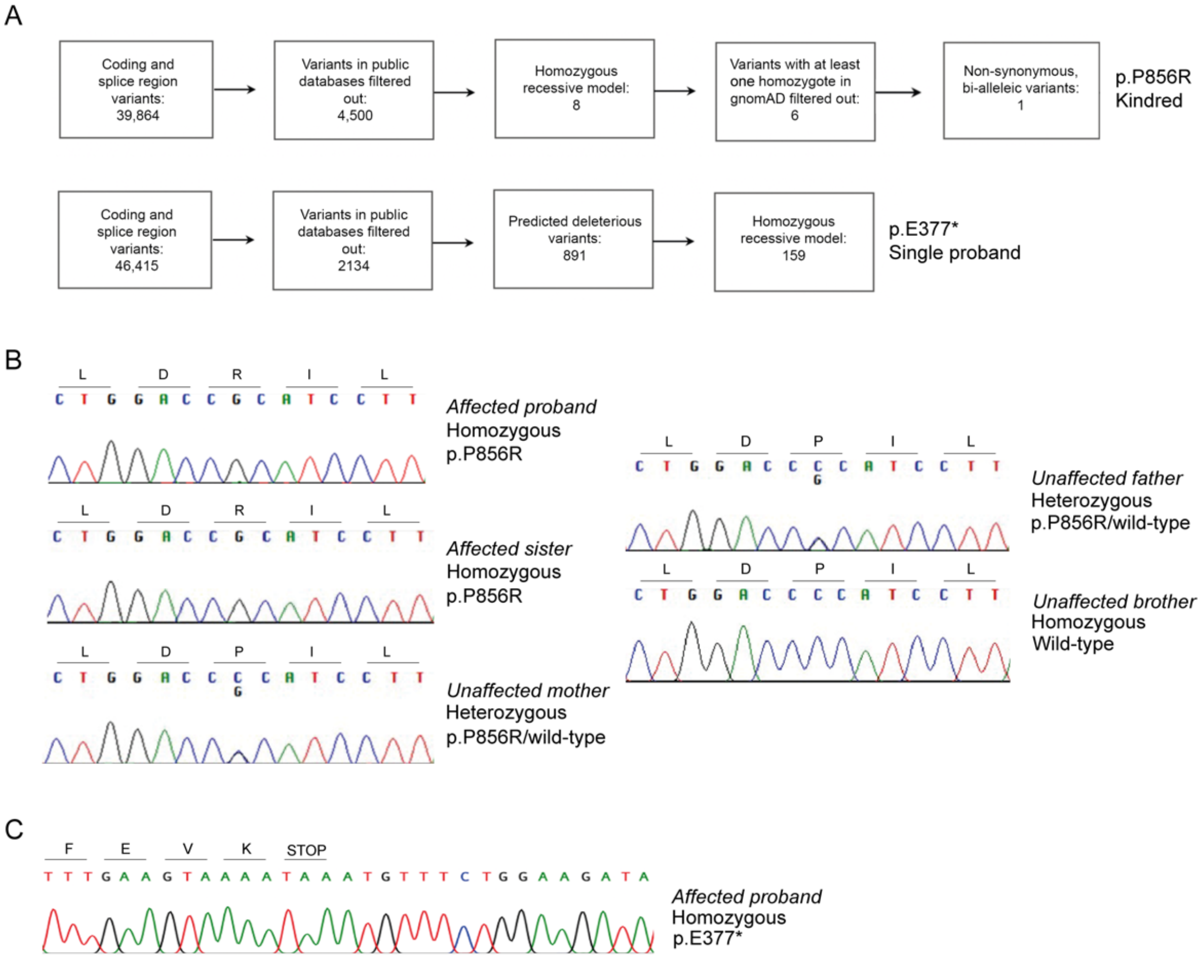

Supplementary Figure 1 *Variant filtering and Sanger sequencing.* A) The variant filtering pipeline was performed using Ingenuity Variant Analysis for both kindred. Variants were initially filtered out where read depth was less than 5 and where changes were present in public databases with a frequency >1%. Subsequently, an autosomal recessive model was applied where affected individuals were homozygous for a given variant and parents were heterozygous. Focussing on non-synonymous changes, loss-of-function, and splice changes lead to the discovery of *YTHDC2* changes in both pedigrees. B) Sanger sequencing of the kindred with the p.P856R variant in *YTHDC2*. C) Sanger sequencing of individual 2 with the p.E377* variant in *YTHDC2*.

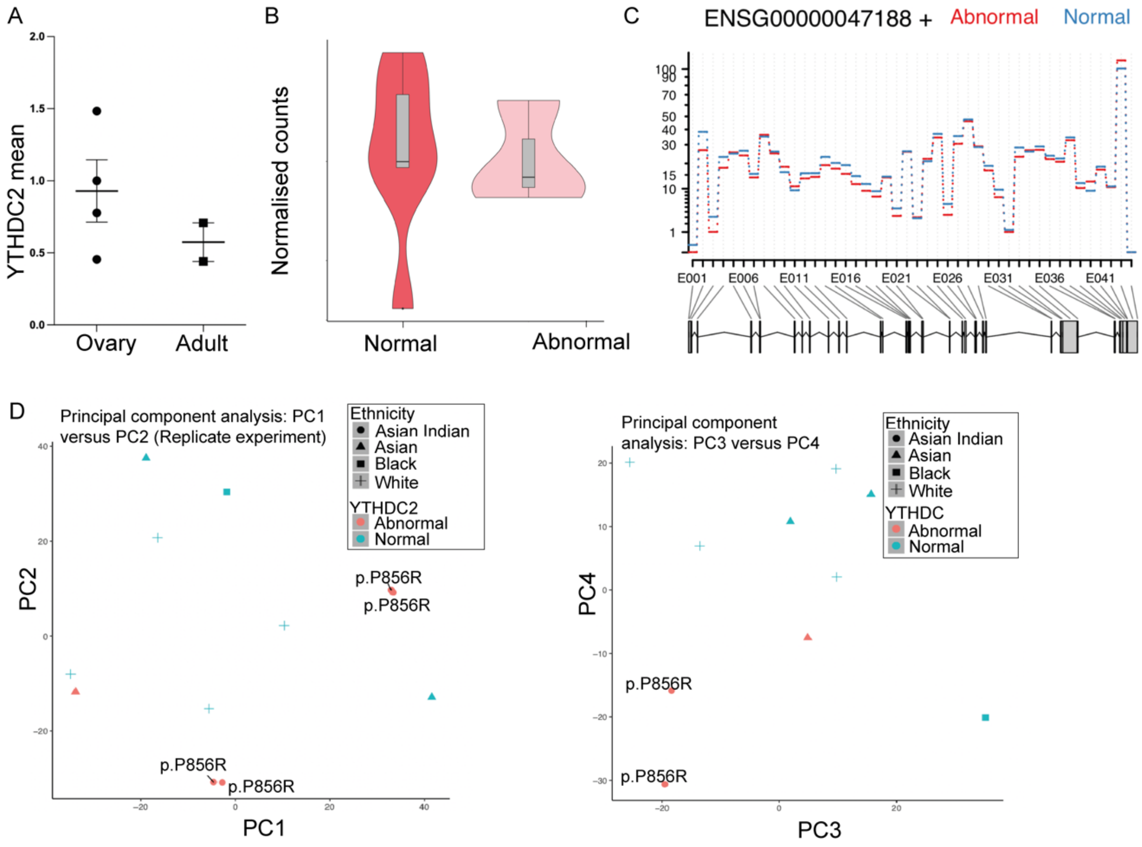

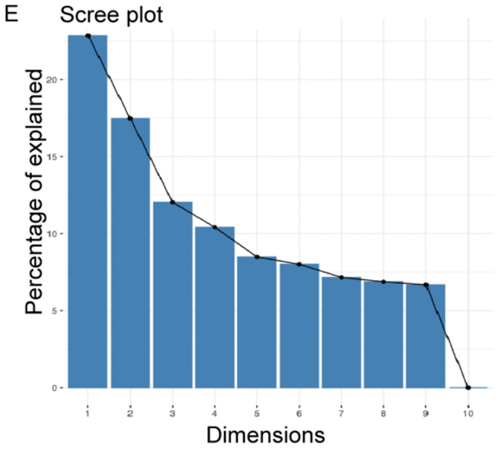

F

Gene name	Log_2FC	p.adj
U2AF1	5.24930375	3.97E-08
PKD1P2	5.11355386	4.29E-05
RNA5-8SN4	3.02601568	0.00258659
ZNF471	0.76035989	0.03690708
NOD2	-0.8258075	0.01120967
TBC1D7	-0.8293306	0.000641
TBC1D7-LOC100130357	-0.8293306	0.000641
TRBV5-5	-1.0637452	0.03690708
TRIM9	-1.3379157	0.00586104
SYCP2L	-1.7189217	0.01120967
C8G	-3.0719716	0.00401232
U2AF1L5	-3.1326508	0.00327299
SMG1P4	-3.589072	0.00051559

Supplementary Figure 2 *YTHDC2 expression in peripheral leukocytes in women with YTHDC2 variants compared to controls.* A) qRT-PCR of *YTHDC2* mean expression (log_2) in adult peripheral leukocytes and in fetal ovary tissue (CS22), demonstrating expression in both tissues. B) Violin plots depicting normalized counts for *YTHDC2* in patients with primary ovarian insufficiency with no known pathogenic variants in *YTHDC2* (n=7) and in patients with confirmed *YTHDC2* variants (n=3). C) Transcript analysis using DEXSeq showed no differences in exon usage or exon splicing between patients and controls (false discovery rate (FDR) <10%) (D) Left panel: Principal component analysis (PCA) of the first (PC1) and second (PC2) principal components including replication of samples with the missense p.P856R changes (normal (n=7) and abnormal (n=5) YTHDC2 samples. Replicates cluster together, demonstrating a stable experimental system for reliable analysis. Right panel: For probity, only one of each technical replicate was included in the further RNAseq analysis. Principal component analysis (PCA) of the third (PC3) and fourth (PC4) principal components when comparing normal (n=7) to abnormal (n=3) YTHDC2 samples, demonstrating segregation of samples with abnormal YTHDC2. (E) Screeplot of variances (eigenvalues) against number of factors (principal components) when comparing normal (n=7) and abnormal (n=3) YTHDC2 samples (no duplicates) showing percentage of variance explained by principal component. (F) Table of top differentially expressed genes comparing normal to abnormal YTHDC2 groups when filtered by a log_2 fold change >0.7 and an adjusted P-value of <0.05. Upregulated genes are more highly expressed in patients with no known *YTHDC2* variants. Downregulated genes are more highly expressed in patients with *YTHDC2* variants.

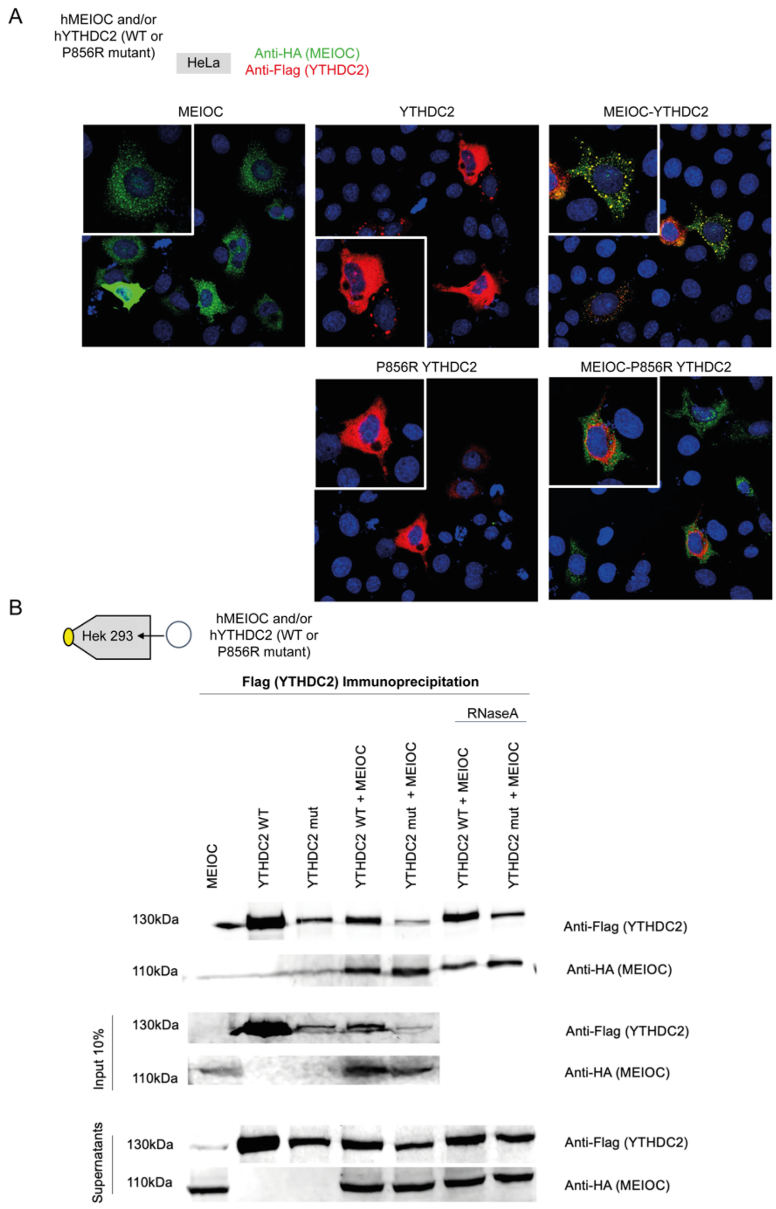

Supplementary Figure 3 *Cellular localization and co-immunoprecipitation of the YTHDC2 p.P856R protein.* A) Transfection of wild-type (WT) or p.P856R FLAG-tagged YTHDC2 alone or with HA-tagged MEIOC into HeLa cells showed that both WT and p.P856R YTHDC2 display similar cytoplasmic localization. In most cells, WT or p.P856R YTHDC2 co-localized with MEIOC (see also Figure 3b), although some perinuclear staining was occasionally observed for YTHDC2. B) Transfection of wild-type (WT) or p.P856R FLAG-tagged YTHDC2 into HEK 293 cells showed both WT and p.P856R YTHDC2associated with MEIOC in a co-immunoprecipitation assay.

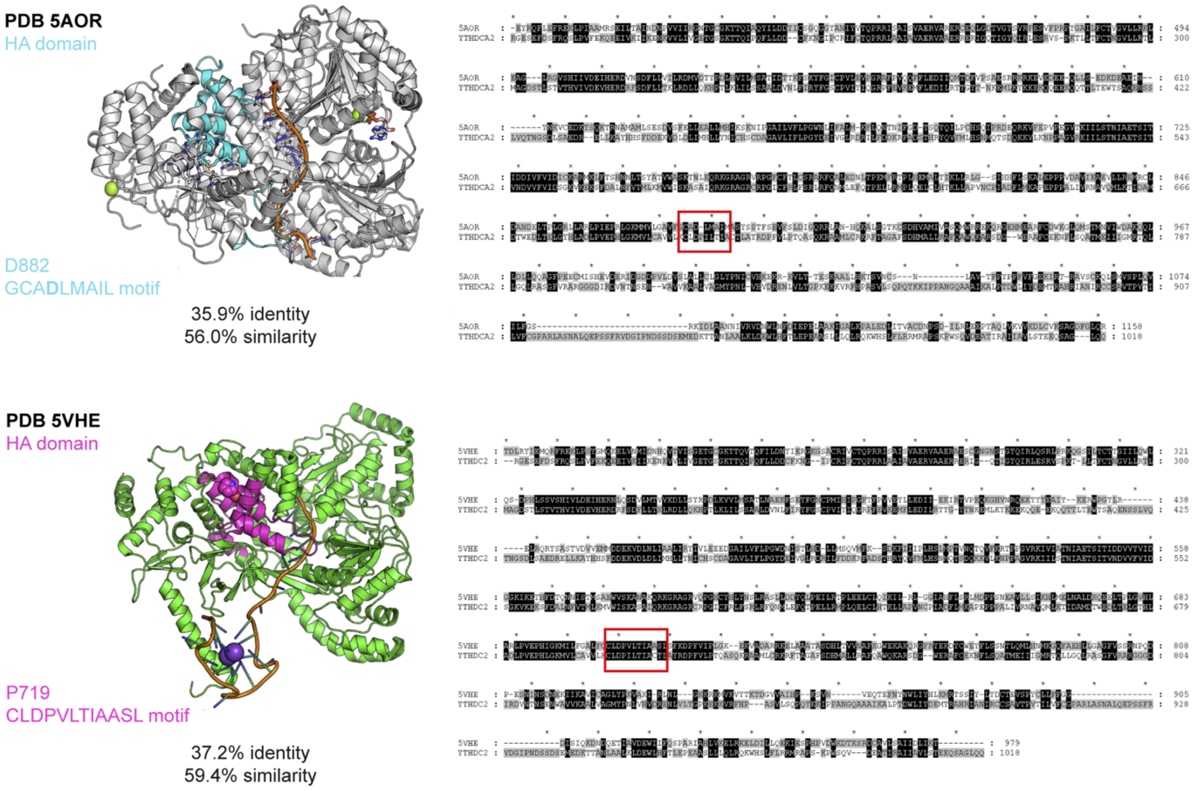

Supplementary Figure 4 *Structural templates and sequence alignment used to generate the initial YTHDC2 models.* The HA domains are highlighted in cyan and magenta for the structures of the MLE RNA ADP AlF4 complex (PDBid 5AOR) (upper panel) and DHX36 in complex with the c-Myc G-quadruplex (PDBid 5VHE) (lower panel). The sequence identities were 35.9 and 37.2%, respectively, while sequence similarities were 56.0 and 59.4%. The motif of interest is shown within a red box.

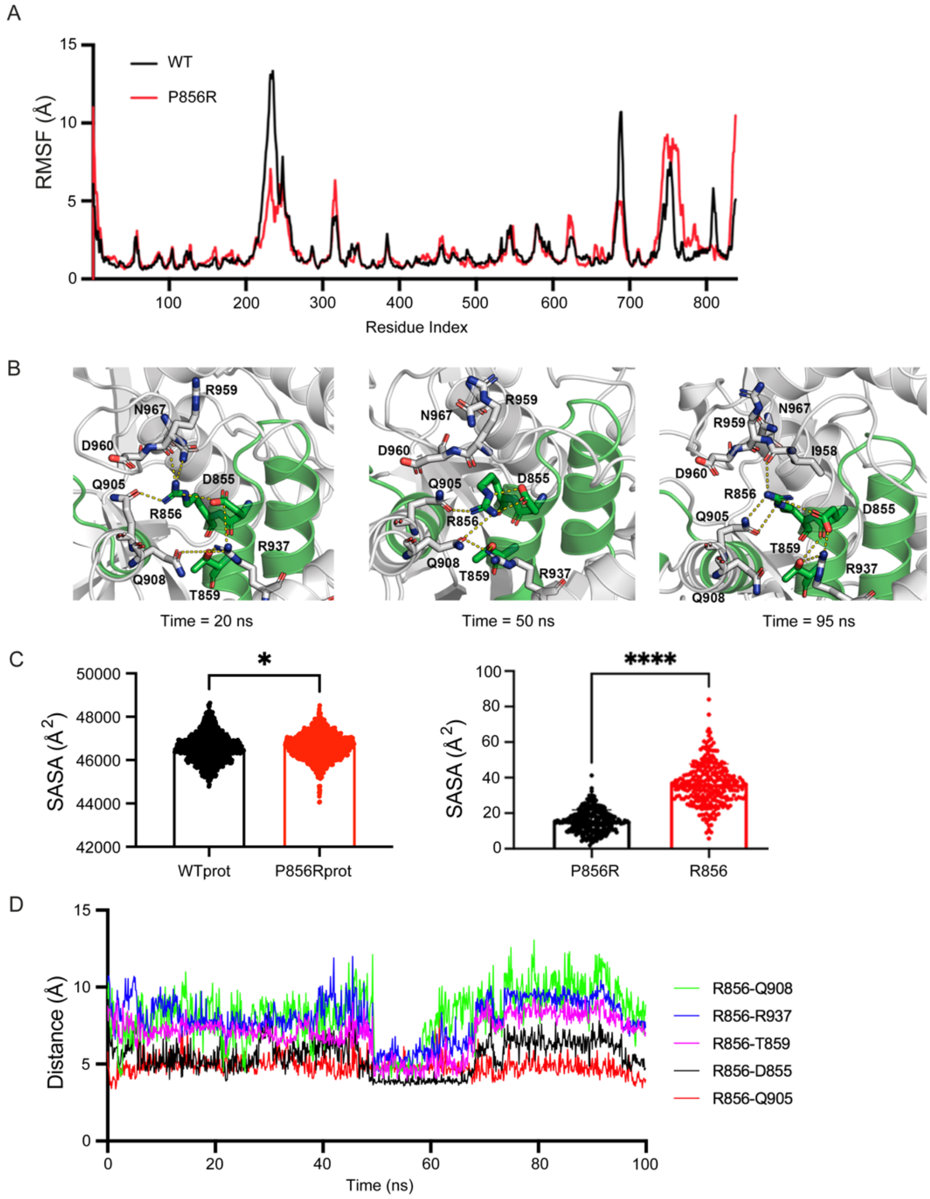

Supplementary Figure 5 *Molecular dynamics trajectory analysis of the YTHDC2 models*. A) Root-mean square fluctuation (RMSF) shows that both proteins have higher movement in the same structural motifs, but wild-type protein is more flexible. B) Representative frames from the simulation of P856R mutant protein showing the hydrogen bond network formed by R856 and neighboring residues, which stabilizes the structure. C) Mutant protein (left panel) and R856 residue (right panel) are more solvent accessible than wild-type counterparts (SASA=solvent accessible surface area). D) Hydrogen-bond distances between R856 and residues D855 (intra-helical salt bridge), Q908, R937, T859, and Q905.

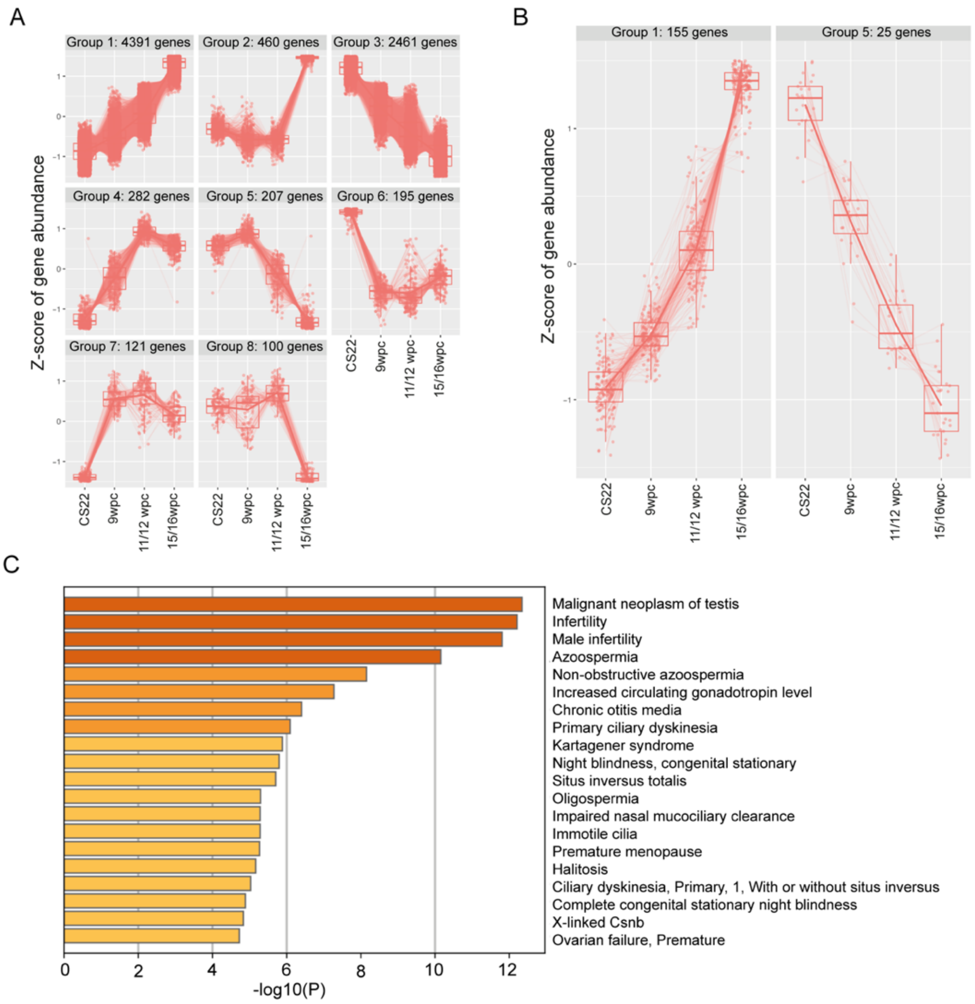

Supplementary Figure 6 *Cluster and pathway enrichment analyses of highly expressed ovary genes* A) Cluster analysis (degPatterns) of all ovary samples (CS22/23 versus 15/16wpc, $log_2FC > 1$, p.adj <0.05). B) Cluster analysis (degPatterns) of all ovary samples with stringent significance testing thresholds (CS22/23 versus 15/16wpc, $log_2FC > 2$, p.adj <1E-25). Meiotic markers from the cluster analysis described in A) were retained within Cluster 1 of this analysis when these stringent clustering criteria were applied. C) Pathway enrichment analysis of genes within Cluster 1 (CS22/23 versus 15/16wpc, $log_2FC > 1$, p.adj <0.05) demonstrating associated disease processes.
